# g-C_3_N_4_ synthesized from NH_4_SCN in a H_2_ atmosphere as a high performance photocatalyst for blue light-driven degradation of rhodamine B†

**DOI:** 10.1039/d0ra02454f

**Published:** 2020-05-22

**Authors:** Shuting Zhang, Guoqiang Li, Liyuan Duan, Hongyu Wang, Yongle Zhao, Yongfa Zhang

**Affiliations:** Key Laboratory of Coal Science and Technology, Ministry of Education and Shanxi Province, Taiyuan University of Technology Taiyuan 030024 China liguoqiang01@tyu.edu.cn

## Abstract

Graphitic carbon nitride (g-C_3_N_4_) was prepared by a simple thermal polymerization method in this work. The effects of precursor type, thermal polymerization temperature, constant temperature time and atmosphere on the crystal structure, morphology, elemental composition, valence distribution, light absorption properties and photocatalytic activity of the prepared photocatalytic materials were investigated. Taking rhodamine B (RhB) as the target degradant, the blue light catalytic activity of the photocatalytic material was studied in detail. The experimental results showed that the final pyrolysis temperature and constant temperature time are positively related to the adsorption characteristics and photocatalytic ability of the prepared materials. In addition, the adsorption capacity and photocatalytic activity of the products obtained in Ar and H_2_ atmospheres are better than those produced in CO and CH_4_, which can be attributed to the combined effect of large specific surface area and structural defects of the materials. The sample's large specific surface area, wide band gap, and excellent photogenerated carrier separation and transfer capabilities make the adsorption performance and photocatalytic performance of the products obtained with ammonium thiocyanate and thiourea as precursors better than those prepared from melamine and dicyandiamide. g-C_3_N_4_ prepared by using ammonium thiocyanate as precursor at 550 °C for 5 h under a hydrogen atmosphere showed the best catalytic activity for the degradation of RhB under blue light. It was demonstrated that g-C_3_N_4_ prepared exhibited good stability and reusability after four repeat experiments. The active components that play major roles in the degradation of RhB described herein were holes and superoxide radicals, which was inferred by free radical trapping experiments. This work provides a theoretical basis for the idea of converting the mixed salts of desulfurization waste liquid containing ammonium thiocyanate into an excellent photocatalyst g-C_3_N_4_ with visible light response.

## Abstract 2

Graphitic carbon nitride (g-C_3_N_4_) was prepared by a simple thermal polymerization method in this work.

## Graphical abstract

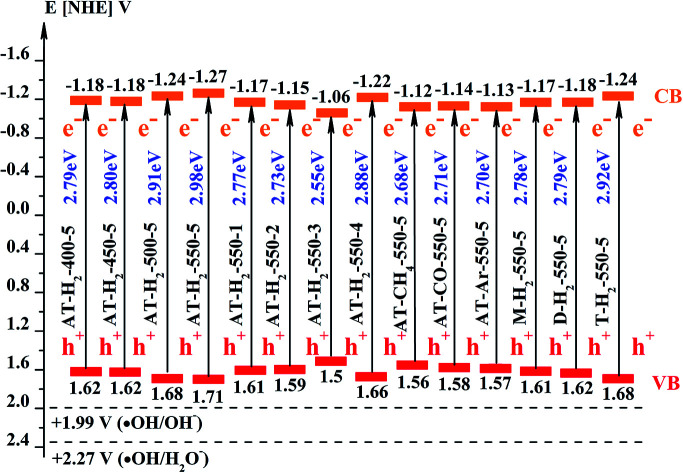

## 1 Introduction

With the rapid development of human society, problems of resources and ecological environment have become increasingly prominent, which has presented a severe test to mankind. As a renewable energy source, solar energy is rich in resources, cheap and clean, and is the basis for the sustainable development of human society.^1^ Therefore, how to efficiently use, transform and store solar energy is an important issue in scientific research. Semiconductor photocatalysis is a green technology that can drive low-density solar energy into high-density chemical energy or directly degrade and purify environmental pollutants by using sunlight to drive a series of important chemical reactions under mild conditions. It has shown great potential in solving problems such as energy shortages and environmental pollution.^2^ From the perspective of practical application of photocatalysis, metal-free photocatalysts have more advantages and have great potential in practical applications. In particular, g-C_3_N_4_ has received extensive attention at home and abroad because of its rich sources, easy availability, good thermal and chemical stability, and excellent performance without secondary pollution to the environment.^3–5^

The development history of g-C_3_N_4_ could be dated back to 1834, when melamine, melam, melem, and melon were discovered by Liebig for the first time;^6^ since then, g-C_3_N_4_ based on triazine ring structure has attracted extensive attention as an ideal precursor for high-temperature and high-pressure synthesis of high-density carbon-nitrogen structures.^7–10^ The research on graphite phase carbonitride has quickly shifted from the initial superhard properties to its photocatalytic applications since Xinchen Wang^11–13^ first reported the excellent performance of g-C_3_N_4_, based on the heptazine ring structure in photocatalysis in 2009, the synthesis research of g-C_3_N_4_ once again become a focus.^14–16^ Pyrolysis of nitrogen-rich organic compounds is the most mature method for synthesizing g-C_3_N_4_ in the preparation of conventional graphite carbonitride^17^ such as melamine,^14,18–24^ dcyandiamide,^25–28^ and ammonium thiocyanate,^29^ melamine chloride (C_3_N_3_Cl_3_),^14^ cyanamide (N_2_H_2_),^30,31^ azide^32^ and guanidine carbonate,^25^*etc.* As a metal-free photocatalyst, g-C_3_N_4_ offers many unique advantages, including a fascinating graphite-like phase layer structure, a suitable band gap of approximately 2.7 eV, excellent stability, nontoxicity, and facile synthesis.^33,34^ In addition, g-C_3_N_4_ products with different morphology structure, polymerization degrees, photoelectric properties and photocatalytic activities can be easily obtained by adding or doping other substances,^35–38^ changing preparation conditions,^39–41^ and coupling with other semiconductors.^5,42,43^

Based on the previous research regarding the pyrolysis of mixed salts obtained from evaporation and concentration of desulfurization waste liquid (rich in ammonium thiocyanate) and the pyrolysis of ammonium thiocyanate in H_2_ atmosphere,^44^ the following work was carried out. In this paper, we chose ammonium thiocyanate, thiourea, melamine and dicyandiamide as nitrogen-containing precursors with different structures to synthesize g-C_3_N_4_ by thermal polymerization. The aim is to systematically compare the different structures and properties of g-C_3_N_4_ produced by different raw materials. In addition to the precursor types, we also studied the effects of pyrolysis end temperature, pyrolysis constant temperature time and pyrolysis atmosphere on the morphology, elemental composition, element valence distribution, specific surface area and pore size distribution of the carbon nitride materials. The activity and stability of the prepared catalysts in photocatalytic degradation of RhB were investigated. The photocatalytic reaction mechanism was also investigated by free radical trapping experiments.

## 2 Experimental

### 2.1 Materials

Ammonium thiocyanate (NH_4_SCN), thiourea (CS(NH_2_)_2_), dicyanamide (C_2_H_4_N_4_), melamine (C_3_H_6_N_6_) and rhodamine B are all obtained from Shanghai Aladdin Biochemical Technology Co., Ltd.

All reagents used in this work are of analytical grade and used as received without further purification. All the water used in this experiment is ultrapure water.

### 2.2 Preparation of g-C_3_N_4_

**Fig. 1:**
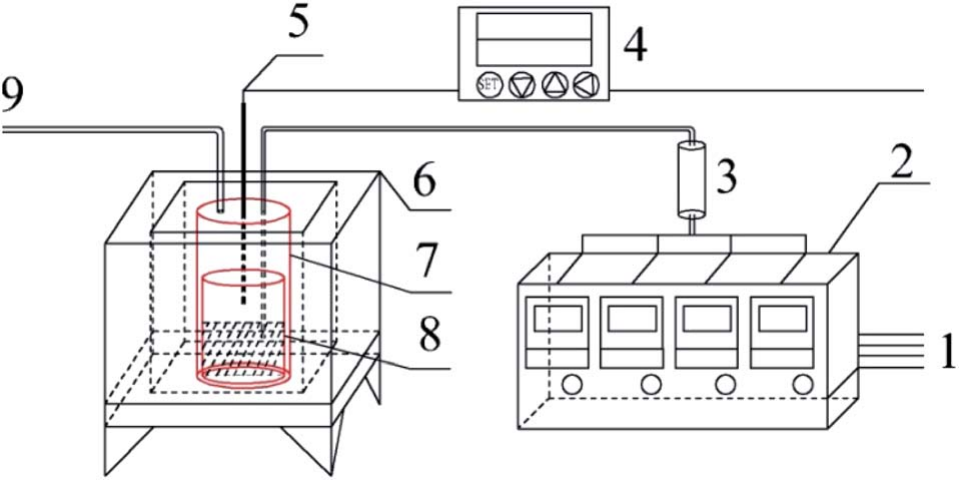
Schematic diagram of the sample preparation device 1-gas source; 2-gas mass flow meter; 3-gas mixing tank; 4-program temperature controller; 5-K type thermocouple; 6-high temperature combustion furnace; 7-stainless steel tank; 8-corundum crucible; 9-gas outlet.

Graphite carbonitride (g-C_3_N_4_) was obtained by thermal polymerization using ammonium thiocyanate, thiourea, dicyandiamide and melamine as raw materials. The schematic diagram of the sample preparation device is shown in Fig. 1. Thirty g of the precursor was placed in the bottom of a cylindrical corundum crucible, which was placed in a stainless steel reaction tank. The reaction tank was placed in a temperature-programmed high-temperature pyrolysis furnace. The precursor was heated from room temperature to the specified temperature (400–550 °C) at a heating rate of 3 °C min^−1^ in a dynamic atmosphere (Ar, H_2_, CH_4_, CO), followed by thermal insulation for 1–5 h. The polymerization tail gas was vented after absorption by sodium hydroxide solution. After the pyrolysis furnace naturally cooled to room temperature, the obtained bulk sample was ground into powder and abbreviated as X–G–*T-t* (where X = AT, T, D, and M represents ammonium thiocyanate, thiourea, dicyandiamide and melamine, respectively; G = Ar, H_2_, CH_4_, and CO refers to the thermal polymerization atmosphere; *T* = 400, 450, 500, and 550 °C refers to the thermal polymerization temperature; *t* = 1, 2, 3, 4, and 5 h represents the thermopolymerization constant temperature time. For example, AT–H_2_-550-5 represents a sample of g-C_3_N_4_ prepared by heating ammonium thiocyanate in H_2_ atmosphere to 550 °C and maintaining the temperature for 5 h).

### 2.3 Analytical methods

The phase analysis of residues was carried out using a Model ULTIMA IV X-ray diffractometer (Rigaku, Japan). It was carried out with an automated X-ray diffractometer using Cu Kα radiation (*λ* = 1.5418 Å), a 40 kV voltage, a 40 mA electrical current, and 2*θ* from 5° to 85°.

The Fourier transform infrared spectra of all samples were obtained using a Vertex 70 Fourier transform infrared spectrometer (FTIR, Bruker, Germany) by the KBr tableting method in the wavelength range of 4000–400 cm^−1^ with a resolution of 4 cm^−1^.

The element at the near surface of the material and its chemical state were determined by X-ray photoelectron spectroscopy (XPS, Thermo ESCALAB 250XI).

The surface morphologies of the samples were characterized with SEM (S4800).

Ultraviolet visible (UV-Vis) diffuse reflectance spectra (DRS) of the samples were measured with a UV-Vis spectrophotometer (U-3900).

The surface area and pore structure of the samples were measured by nitrogen adsorption–desorption testing on a specific surface and pore size analysis instrument (3H–2000PS2). The nitrogen sorption isotherms were measured at 77 K with a 3H–2000PS2 analyzer after the samples were degassed in a vacuum at 190 °C for 10 h.

The elements such as C, H, N, and S in the prepared samples were quantitatively analyzed by the German Elementar analyzer (EA).

### 2.4 Photo catalytic activity evaluation

**Fig. 2:**
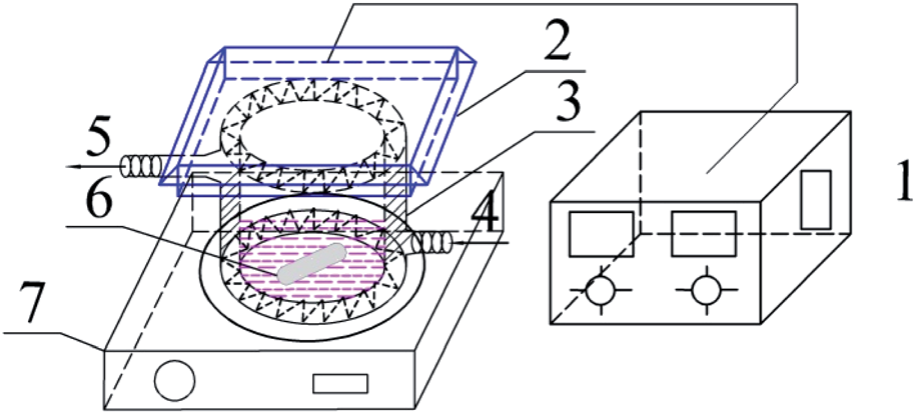
Schematic diagram of the experimental device for photocatalytic degradation of RhB 1-light source regulator; 2-LED blue light source; 3-photocatalytic reaction beaker; 4-cooling water inlet; 5-cooling water outlet; 6-rotor; 7-magnetic stirrer.

**Fig. 3:**
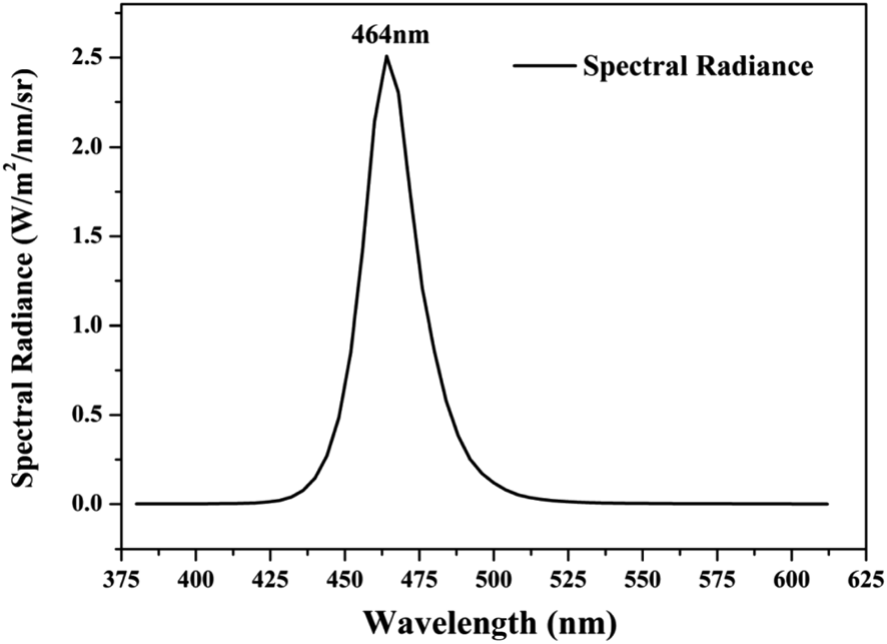
Spectrum curve of blue LED light source.

Photocatalytic activities of prepared samples were illustrated by the photodegradation of rhodamine B under visible light irradiation using a 10 W LED with a maximum emission line peak at 464 nm (L100-100-20, 425–525 nm, > 50 000 Lux, Dongguan Yingshi Electronic Technology Co., Ltd., China). The schematic diagram of the photocatalytic evaluation experimental device is shown in Fig. 2. Fig. 3 shows the spectrum of the LED light source. In the photocatalytic test, 0.3 g of the prepared sample was dispersed in 300 mL of a rhodamine B solution having a concentration of 10 mg L^−1^. The suspension was magnetically stirred in the dark for 90 minutes to ensure an adsorption/desorption equilibrium between rhodamine B and the photocatalyst. After this, the reactant system was placed under blue light irradiation: during the process, 6 mL of suspension was removed from the reactor at a given time interval and centrifuged for 5 minutes at a rate of 10 000 rpm to achieve solid–liquid separation. The concentration of RhB was determined by measuring the absorbance of the solution at a wavelength of 553 nm after centrifugation using a UV-vis spectrometer (UV-1500 Shanghai Macy Instrument Co., Ltd.), and then converting it by a standard curve.

The degradation rate of RhB can be calculated according to formula (1):1
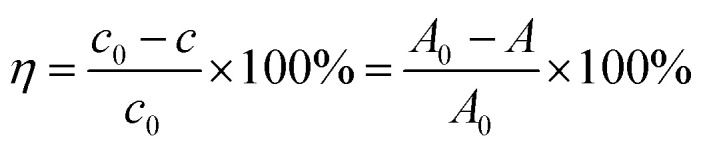
where: *η* is the efficiency of photocatalytic degradation of RhB by the catalyst; *c*_0_ is the concentration of the original solution; *c* is the concentration of samples taken during photocatalytic experiments; *A*_0_ is the absorbance value of the original solution at 553 nm; *A* is the absorbance value at 553 nm measured after centrifugation of the sample.

## 3 Results and discussion

### 3.1 Morphology and structural characterization of g-C_3_N_4_

#### 3.1.1 Scanning electron microscopy analysis results

**Fig. 4:**
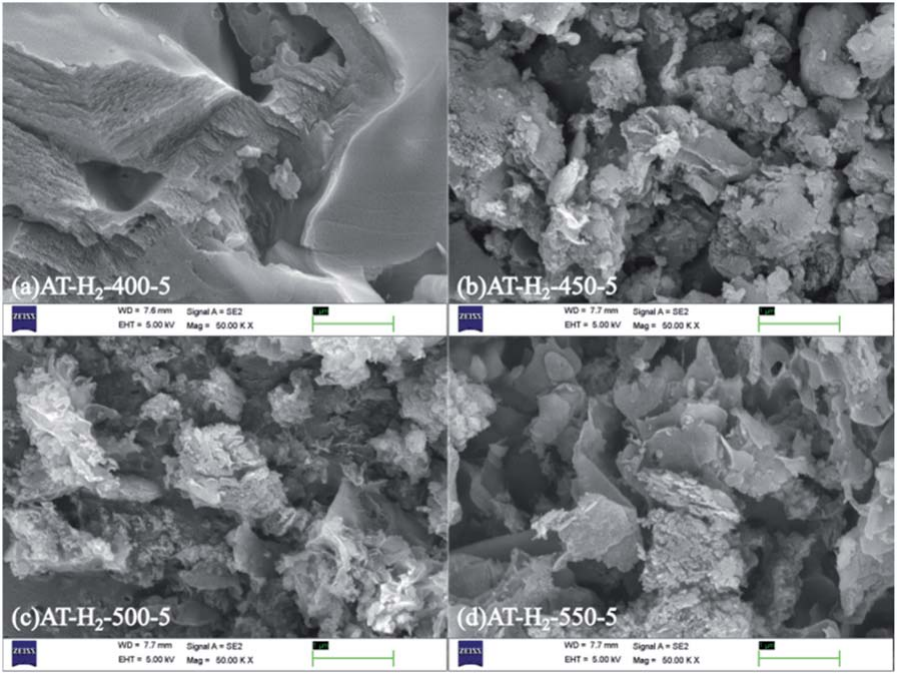
SEM images of g-C_3_N_4_ obtained by pyrolysis of NH_4_SCN at 400–550 °C in H_2_ for 5 h.

Fig. 4 shows the local morphology and structure of the bulk g-C_3_N_4_ synthesized by thermal polycondensation of ammonium thiocyanate at different temperature. As the picture shows the products of ammonium thiocyanate obtained at 400 °C are obviously thick-layer porous blocks, and the products at 450 °C are composed of larger irregular particles and some layered structures. With the increase of thermal polymerization temperature, the product bulk decreases. CN materials prepared at 500 °C are mainly composed of thin sheets and particles, and pore size structure is gradually formed at the same time.^45^ The lamellar structure gradually appeared of 550 °C products, the lamellar thickness became thinner and the structure became more and more fluffy. It can be seen that increasing the reaction temperature is helpful for the formation of smaller and thinner porous CN nanomaterials.

**Fig. 5:**
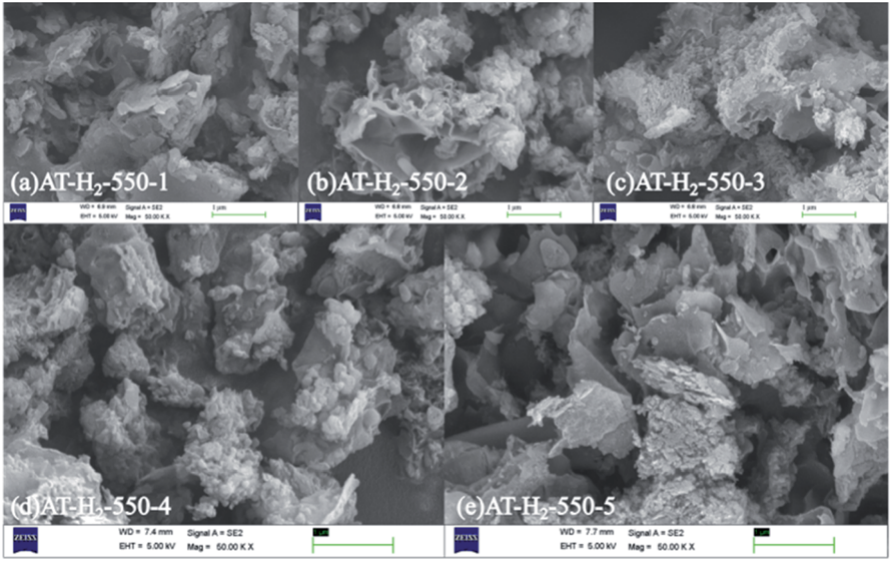
SEM images of g-C_3_N_4_ obtained by pyrolysis of NH_4_SCN at 550 °C in H_2_ for 1–5 h.

Fig. 5 shows the SEM images of g-C_3_N_4_ obtained by pyrolysis of ammonium thiocyanate at 550 °C in H_2_ atmosphere for 1, 2, 3, 4 and 5 h, respectively. It can be observed that, with the increase of the polymerization time, the products of 1 h exhibit some lamellar structures attached to the agglomerated particles; the products of 2 h and 3 h have similar apparent structures, similar to dry tremella and have a fine porous structure. There are some bubbly structures besides auricularia for the 4 h products, which are attributed to a large number of gases produced during the pyrolysis of ammonium thiocyanate, and porous nanosheets are formed in the 5 h product. g-C_3_N_4_ exhibits a two-dimensional nanosheet structure consisting of small plates with irregularly shaped pleats that provide larger specific surfaces and more reactive sites.

**Fig. 6:**
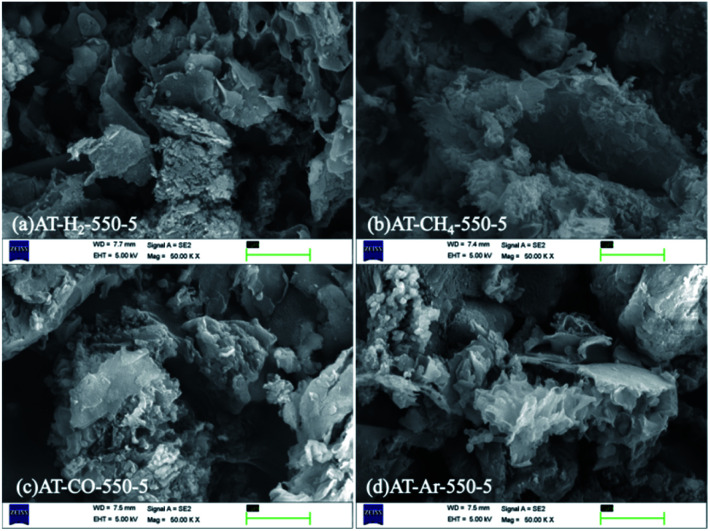
SEM images of products obtained by thermal polymerization of NH_4_SCN at 550 °C in different atmospheres.

The SEM images (Fig. 6) of products obtained by thermal polymerization of ammonium thiocyanate at 550 °C in four different atmospheres showed that the products produced in the H_2_ atmosphere were nanosheets. The products in the CH_4_ atmosphere and in Ar and CO atmospheres were similar to those tremella-like thin-layer aggregates, and the products in the CH_4_ atmosphere looked more fluffy.

**Fig. 7:**
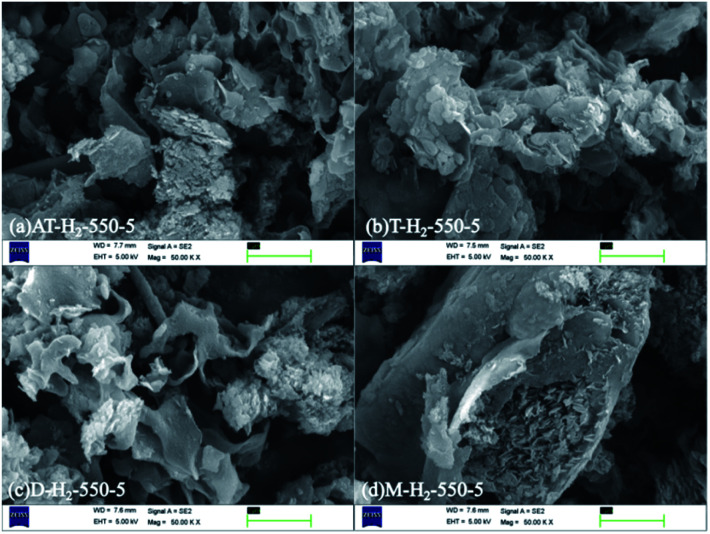
SEM images obtained by thermal polymerization of different precursors at 550 °C for 5 h in H_2_ atmosphere.

Graphite phase carbon nitride SEM images in Fig. 7 obtained by thermal polymerization of ammonium thiocyanate, thiourea, dicyandiamide and melamine at 550 °C in H_2_ atmosphere showed that thiourea products were stacked as cornflake-shaped nanosheets; dicyandiamide products were curved flakes and tremella-like; melamine thermal polymerization products were stacked as nanosheets and small and thin sheets. The sample prepared from melamine has a very smooth block surface with a large block size. The above obvious morphological and dimensional differences can be attributed to the inherent properties of different nitrogen-rich precursors, such as melting point, amino content, degree of polymerization, and preferential reaction pathways.^45^ Due to the release of NH_3_ and sulfur species (such as H_2_S and CS_2_) during the decomposition of ammonium thiocyanate and thiourea, the connectivity mode and topological structure of g-C_3_N_4_ are changed.^51^

#### 3.1.2 Specific surface area and pore size distribution analysis

**Fig. 8:**
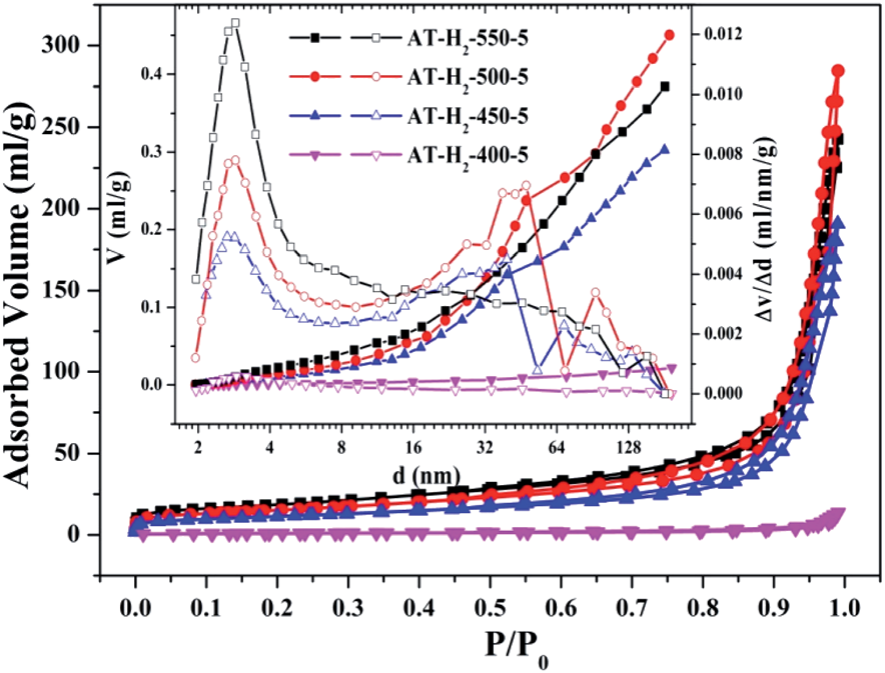
Nitrogen adsorption–desorption isotherms and the corresponding pore size distribution curves (inset) of the samples prepared at different temperature.

**Fig. 9:**
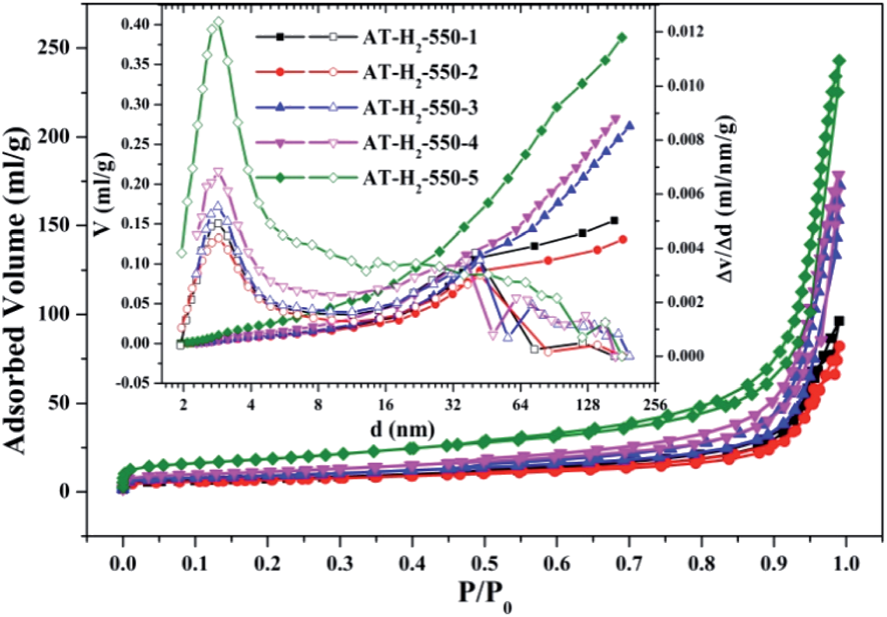
Nitrogen adsorption–desorption isotherms and the corresponding pore size distribution curves (inset) of the samples prepared in different temperature maintenance time.

**Fig. 10:**
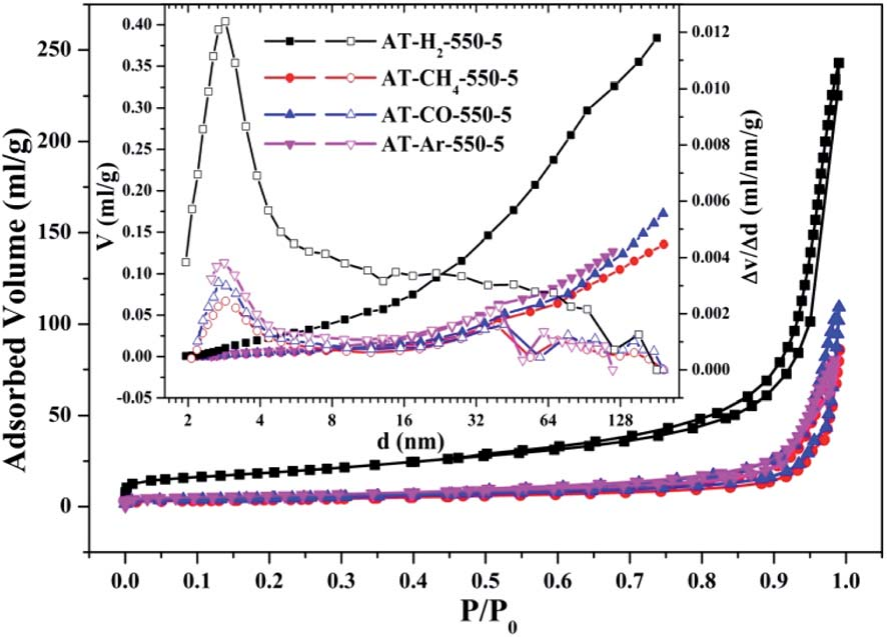
Nitrogen adsorption–desorption isotherms and the corresponding pore size distribution curves (inset) of the samples prepared in different atmospheres.

**Fig. 11:**
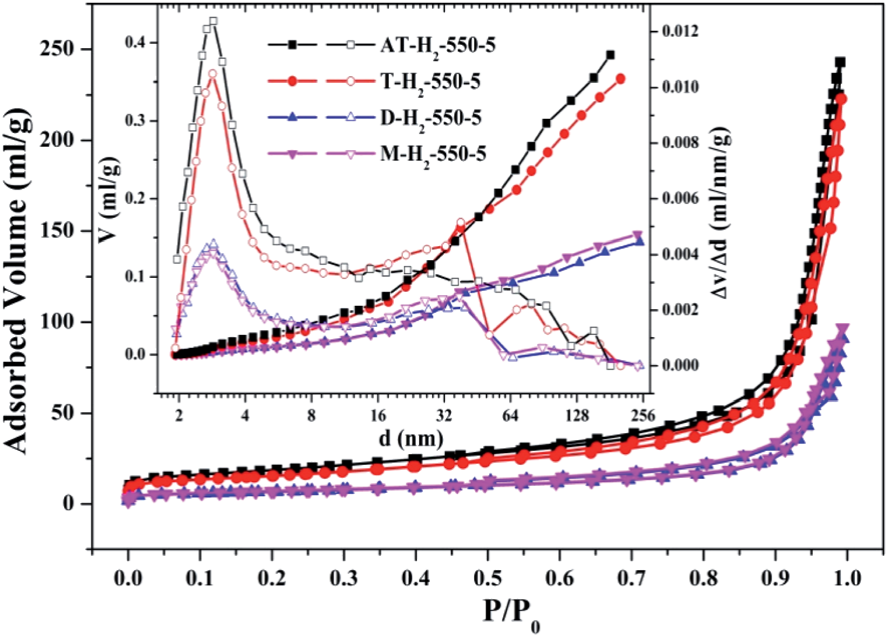
Nitrogen adsorption–desorption isotherms and the corresponding pore size distribution curves (inset) of the samples prepared by different precursors.

To characterize the specific surface area of the obtained sample, the photocatalysts were tested and analyzed by N_2_ adsorption–desorption isotherms. Some studies have shown that the macroscopic and mesoporous properties of the catalyst have an effect on the scattering of light. Highly porous materials can produce more photoactivated electrons.^46,47^Fig. 8–11 shows the nitrogen adsorption analysis curves and the Barrett–Joyner–Halenda (BJH) pore size distribution curves of the g-C_3_N_4_ samples prepared under different conditions. As illustrated in Fig. 8–11, except for the samples prepared by pyrolysis at a final temperature of 400 °C, the adsorption desorption curves of all materials at high relative pressure (0.5–1.0) display the typical type IV isotherms (classified by BDDT) and type H3 hysteresis loops, indicating the formation of mesopores and macropores in the sample. The isotherms do not have clear saturated adsorption platforms, which indicates that the pore structure is very irregular. The reflected holes include flat slit structures and cracks. The illustrations in Fig. 8 show a large range of pore sizes (2–230 nm), further confirms the existence of mesopores and macropore structures in the material.

The specific surface area (*S*_BET_, m^2^ g^−1^), total pore volume (*V*_pore_, cm^3^ g^−1^), and BJH average pore width (*D*_pore_, nm) are shown in Table S1.† The data in the table clearly show that the specific surface area of the product obtained by thermal polymerization of ammonium thiocyanate at 550 °C under H_2_ increases from 24.05 m^2^ g^−1^ to 65.88 m^2^ g^−1^ with the pore volume gradually increased from 0.13 cm^3^ g^−1^ to 0.39 cm^3^ g^−1^ as the thermal polymerization constant time is extended from 1 h to 5 h. It means that with the extension of the pyrolysis isothermal time and as the degree of polymerization of the resulting product increases, the specific surface area of the product also increases significantly, which is advantageous for improving the photocatalytic activity of the prepared materials. Similarly, as the thermal polymerization temperature gradually increased from 400 °C to 550 °C, the structure of the obtained material changed from a bulk structure to a lamellar structure, and the degree of heat shrinkage of the sample was enhanced. At the same time, the specific surface area also increases, which further increases the number of active sites on the sample surface.

The specific surface areas of pyrolysis products produced in four different atmospheres are ranked in the order of AT–H_2_-550-5 > AT–Ar-550-5 > AT–CO-550-5 > AT–CH_4_-550-5. This should be attributed to the difference in pyrolysis characteristics of ammonium thiocyanate under four atmospheres, including the generation of gas-phase products and differences in pyrolysis reaction rate.

The yields of g-C_3_N_4_ prepared from four precursors such as ammonium thiocyanate, thiourea, melamine, and dicyandiamide were 9.21%, 7.25%, 40.5%, and 31.1%, respectively. The types and yields of pyrolysis products of AT and T are significantly more than those of D and M, resulting in AT and T products exhibiting specific surface areas of 65.66 m^2^ g^−1^ and 54.74 m^2^ g^−1^ which are significantly greater than 23.94 m^2^ g^−1^ and 24.50 m^2^ g^−1^ of D and M products, which fits well with the results of the photodegradation experiment (Fig. 37). It can be seen that there is a positive correlation between the specific surface area of the prepared sample and the photocatalytic degradation rate,^48^ which can be interpreted as a larger specific surface area can provide more reaction sites for the reactants.

#### 3.1.3 X-ray diffraction analysis results

**Fig. 12:**
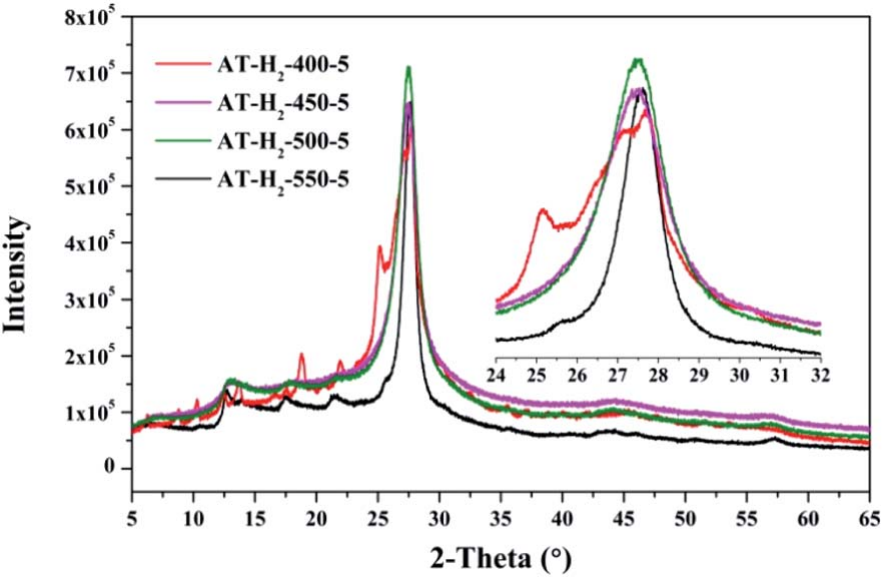
XRD patterns of synthesized g-C_3_N_4_ under different conditions.

**Fig. 13:**
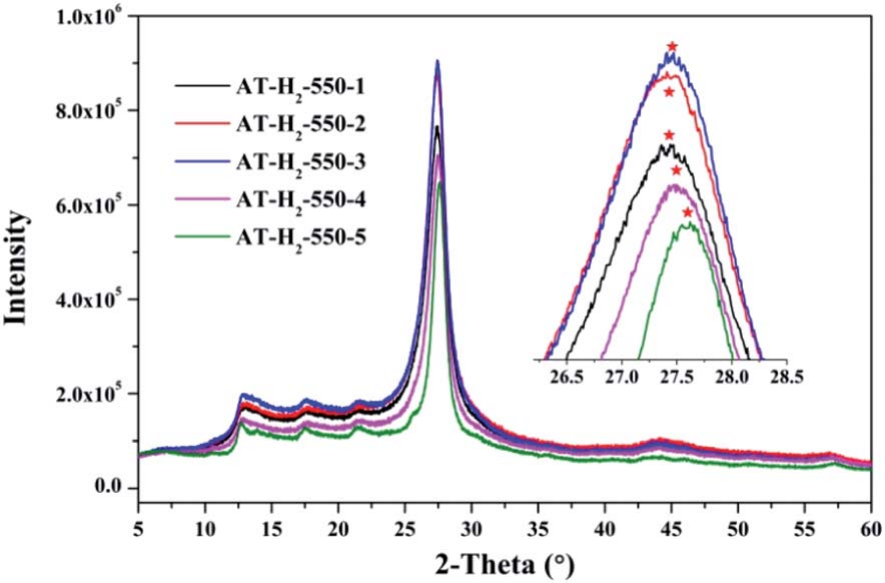
XRD patterns of g-C_3_N_4_ prepared in different temperature maintenance time.

**Fig. 14:**
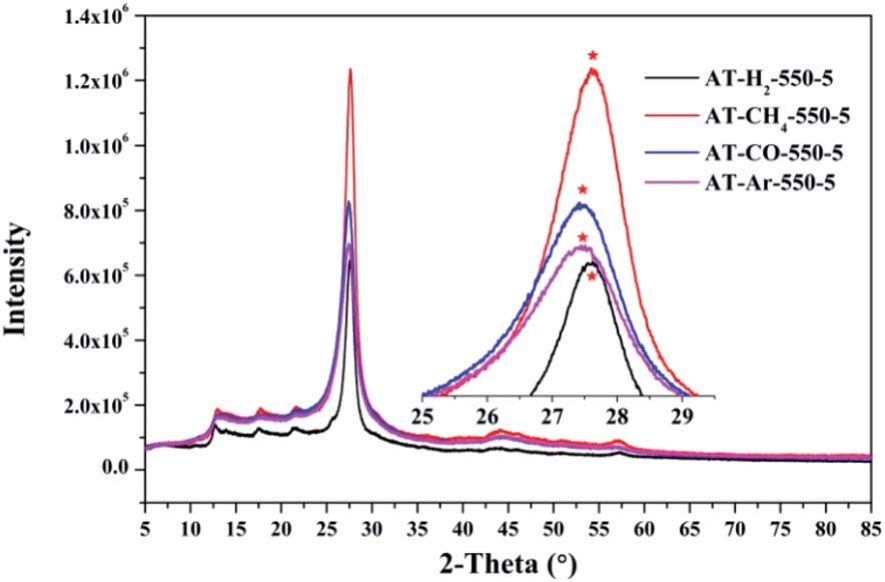
XRD patterns of synthesized g-C_3_N_4_ prepared in different atmospheres.

**Fig. 15:**
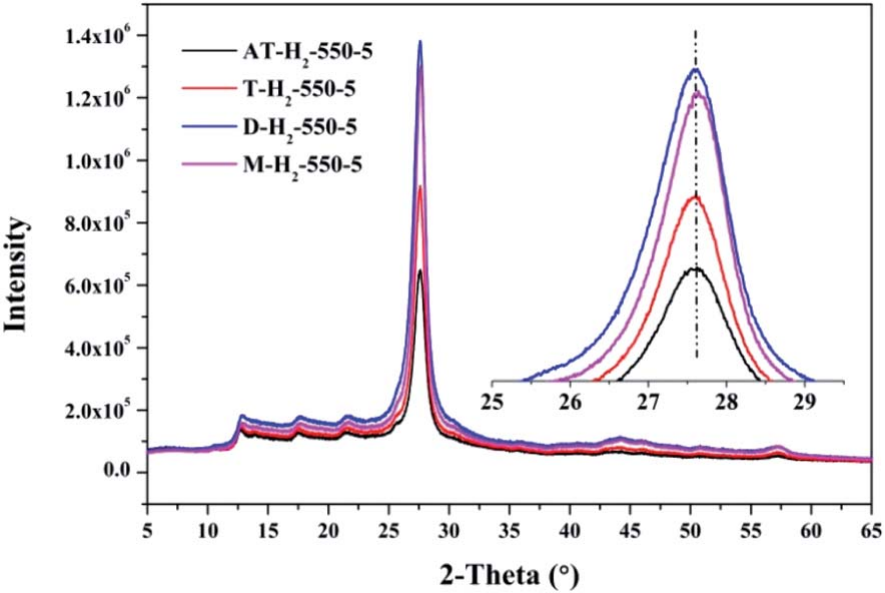
XRD patterns of g-C_3_N_4_ prepared by different precursors.

Fig. 12–15 show the XRD patterns of the produced samples synthesized by various precursors at various temperatures in various atmospheres. As shown in figures, two typical diffraction peaks at nearly 13.0° and 27.0° are observed in the XRD pattern of prepared samples, which are attributed to the in-plane repeated tri-*s*-triazine unit (001) and the stacking of the conjugated aromatic systems (002), respectively, indicating the successful synthesis of g-C_3_N_4_ by thermal polymerization of different precursors under different pyrolysis conditions.^49^ In addition, the characteristic diffraction peaks at approximately 17.5° and 22.5° can be assigned to the (100) and (101) crystal planes of the sample.

As shown in Fig. 12, with increasing of the heating temperature from 400 °C to 550 °C, the diffraction peak assigned to (002) shifted from 27.3° to 27.62°, indicating that increased heating temperature led to the damage of dense stacking between the conjugated aromatic systems. Note that g-C_3_N_4_ prepared at 400 °C contains an amount of impurity phase originating from incomplete decomposition of the ammonium thiocyanate.^44^

As can be seen in Fig. 13, when the constant temperature time is 1, 2, 3, 4, 5 h, the corresponding (002) peak positions of g-C_3_N_4_ prepared by ammonium thiocyanate are 27.44°, 27.45°, 27.47°, 27.50°, 27.62°. According to the Bragg equation, with the increase of the pyrolysis constant temperature time, the spacing of the graphite phase carbon nitride layer obtained continues to shrink, and the crystallinity of the obtained sample increases.

Similarly, the pyrolysis atmosphere also has an effect on the crystallinity of the product. As shown in Fig. 14, the XRD peaks (002) of the products obtained by ammonium thiocyanate in the Ar, CO, CH_4_ and H_2_ atmospheres at 550 °C for 5 h are located at 27.45°, 27.46°, 27.63° and 27.63°, respectively. This indicates that the interlayer spacing of the products obtained in the H_2_ or CH_4_ atmosphere is smaller than that of the products obtained in the Ar or CO atmosphere, which may be the result of the hydrogen bonding in the interlayer structure of the material.

The XRD patterns (Fig. 15) of the pyrolysis products obtained by different precursors at 550 °C for 5 h under H_2_ atmosphere showed that there were no significant differences in the g-C_3_N_4_ characteristic diffraction peak positions prepared by the four precursors. The ranked intensities of the diffraction peaks were AT–H_2_-550-5 < T–H_2_-550-5 < M–H_2_-550-5 < D–H_2_-550-5.

#### 3.1.4 Fourier transform infrared spectroscopy results

**Fig. 16:**
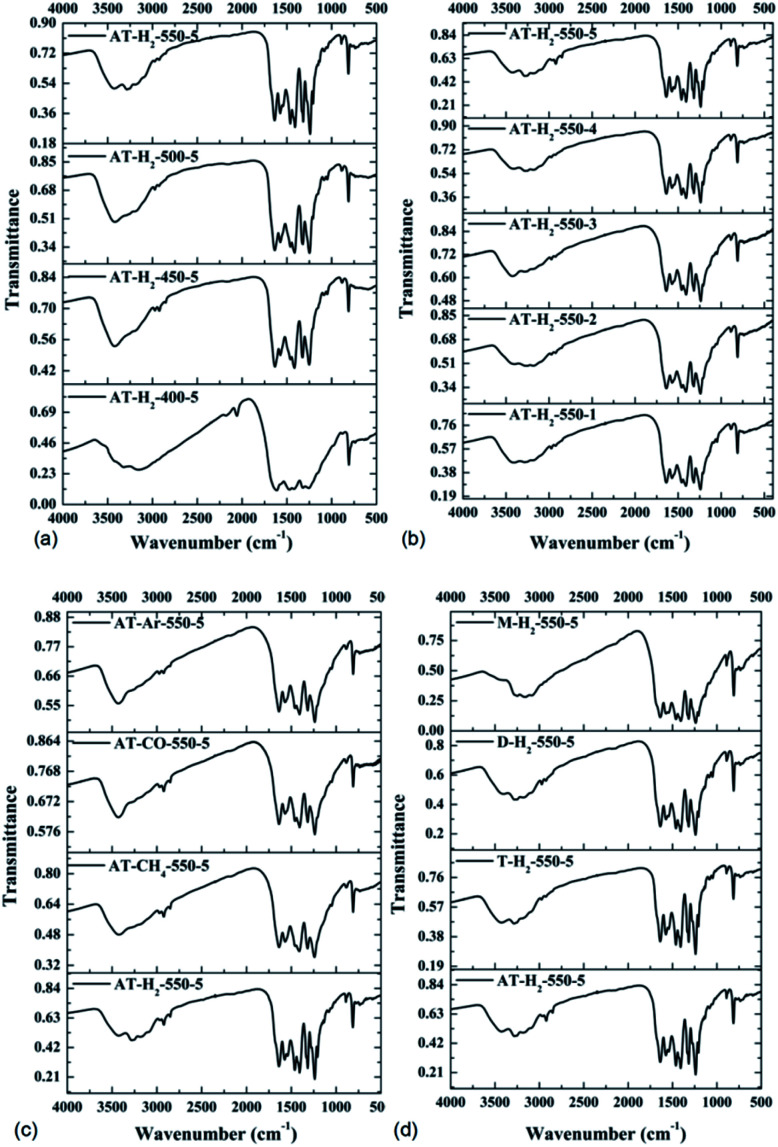
FTIR spectra of synthesized g-C_3_N_4_ prepared (a) at different temperaure (b) in different temperature maintenance time (c) in different atmospheres (d) by different precursors.

FTIR spectra were used to identify the chemical functional groups of prepared samples. Fig. 16 is the FTIR spectrum of the pyrolysis products obtained under different preparation conditions: as shown in the figure, the sharp peak at approximately 801 cm^−1^ is due to the stretching vibration of the triazine/*s*-triazine ring, the peaks in the region from 1100 to 1600 cm^−1^ can be attributed to the characteristic stretching vibrations of C–N and C=N heterocycles, and the wider absorption signals in the range of 3000–3700 cm^−1^ are attributable to the stretching vibrations of N–H and O–H groups, exhibiting partially uncondensed amino groups and physically adsorbed water.^50^

It is worth noting that for samples obtained at low temperatures of 400 °C and 450 °C (Fig. 16(a)), incomplete polymerization of NH_4_SCN results in weak vibrational modes of heptazine units. The characteristic band at 2165 cm^−1^ corresponded to the C≡N stretching mode.^50^ As the pyrolysis temperature increases, the FTIR characteristic peak of the obtained product in the range of 1100–1600 cm^−1^ gradually becomes sharper and the peak intensity increases, indicating that the product polymerization degree is enhanced. It can be seen from Fig. 16(b, c and d) that the constant temperature time, pyrolysis atmosphere and pyrolysis precursor have no distinct influence on the FTIR characteristic peak position or intensity of the obtained product.

#### 3.1.5 Elemental analysis results

**Fig. 17:**
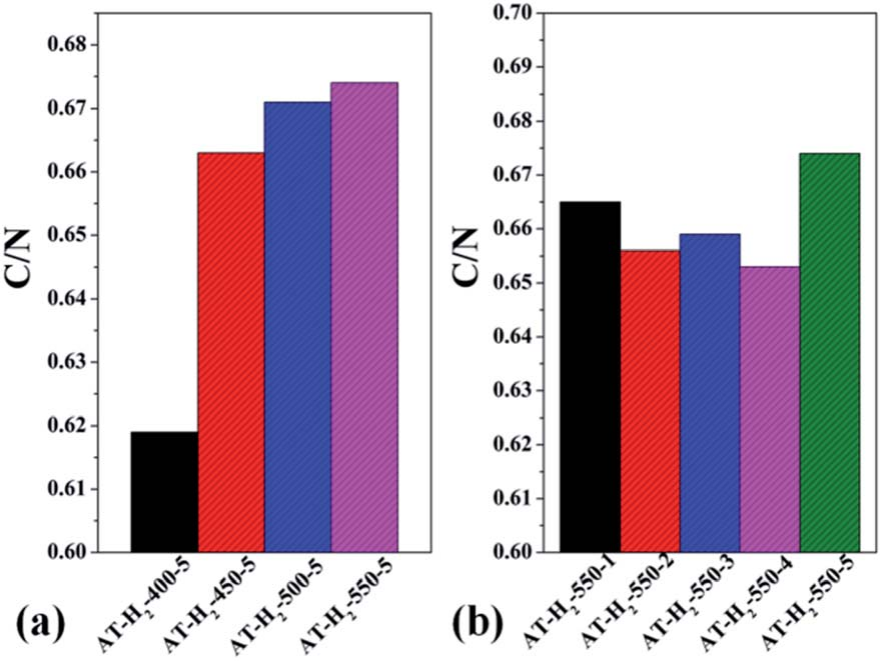
Molar ratios of C/N in the products obtained (a) at different temperaure (b) in different temperature maintenance time.

**Fig. 18:**
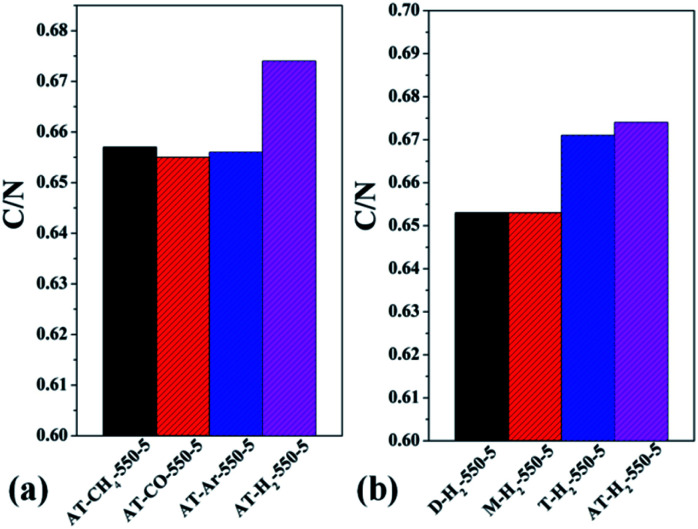
Molar ratios of C/N in the products obtained (a) in different atmospheres (b) by different precursors.

Table S2† gives the C, H, N, S elemental analysis data and C/N molar ratio of the prepared samples. Fig. 17 and 18 show the bar graphs for the molar ratio of carbon to nitrogen in the products obtained under each preparation condition. As seen from the figure, the sample corresponding to a maximum C/N ratio of 0.674 is AT–H_2_-550-5, which performed best in photocatalytic oxidation of RhB degradation (Fig. 33–36) although the C/N ratio is less than the ideal value of 0.75. It can be seen from Fig. 17(a) that the C/N ratio of the pyrolysis product gradually increases from 0.619 to 0.674 as the final temperature of pyrolysis increases from 400 °C to 550 °C. The C/N ratios of the prepared samples have no obvious regularity with the extension of the constant temperature time as shown in Fig. 17(b). It can also be seen that the C/N ratios of the products prepared under the atmospheres of CH_4_, CO and Ar are essentially the same, at approximately 0.656 as shown in Fig. 18(a). As shown in Fig. 18(b), the C/N ratios of the products prepared by using dicyandiamide and melamine as precursors are also close, and the C/N ratio of the product prepared from thiourea as the precursor is slightly smaller than that prepared by using ammonium thiocyanate.

#### 3.1.6 X-ray photoelectron spectroscopy results

**Fig. 19:**
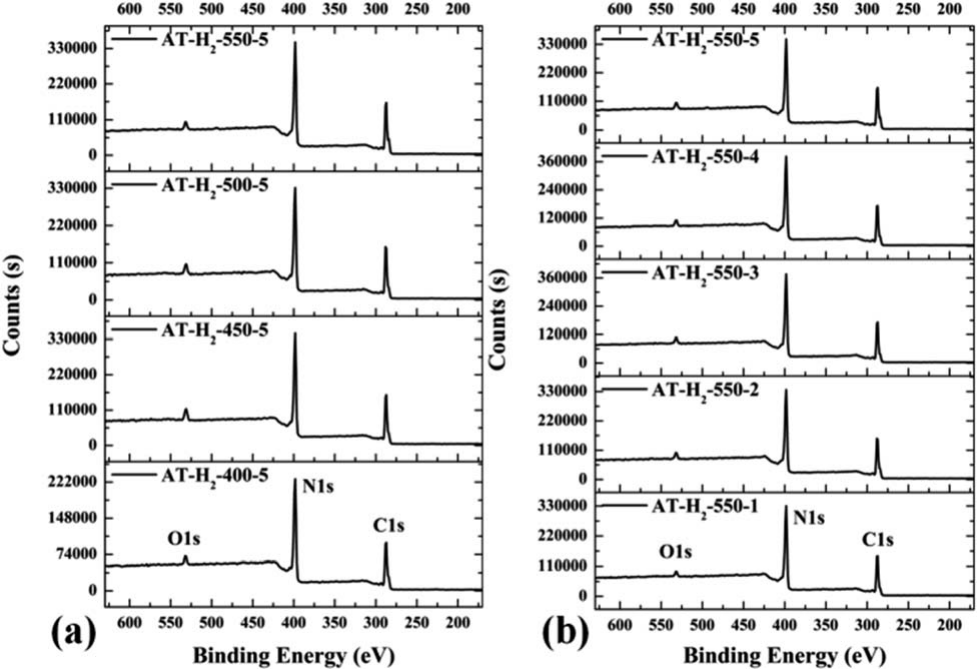
XPS survey spectrum for samples prepared (a) at different temperature (b) in different temperature maintenance time.

**Fig. 20:**
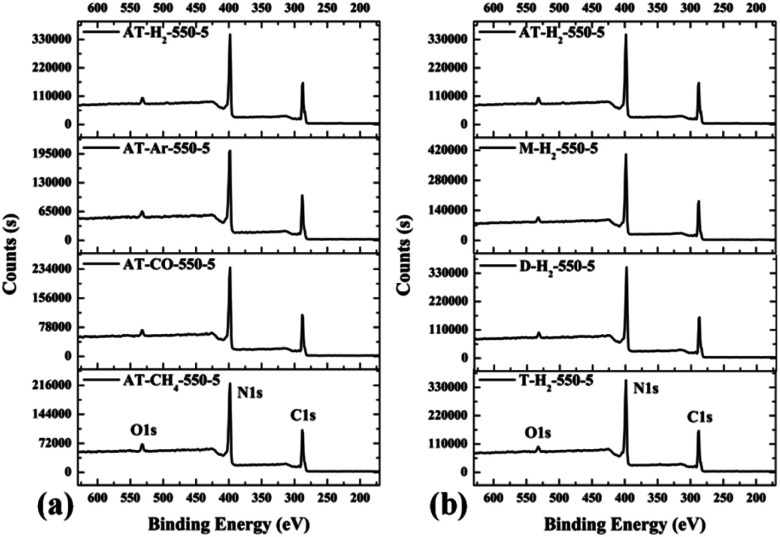
XPS survey spectrum for samples prepared (a) in different atmospheres (b) by different precursors.

**Fig. 21:**
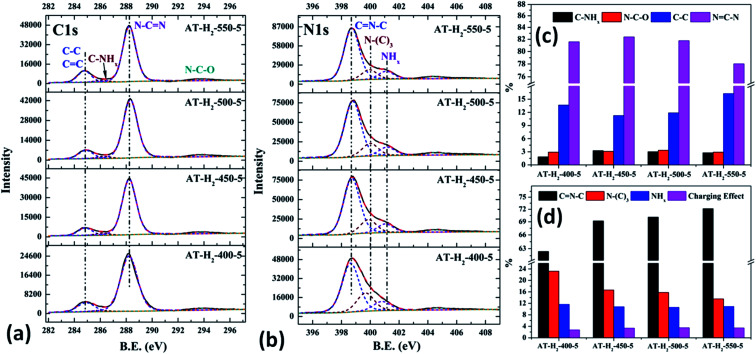
The high-resolution XPS spectra of C 1s (a), N 1s (b) and proportion of each state of C (c) and N (d) element for samples prepared at different temperature.

**Fig. 22:**
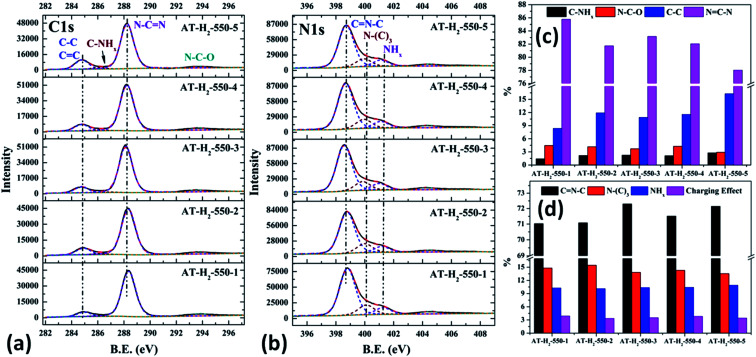
The high-resolution XPS spectra of C 1s (a), N 1s (b) and proportion of each state of C (c) and N (d) element for samples prepared in different temperature maintenance time.

**Fig. 23:**
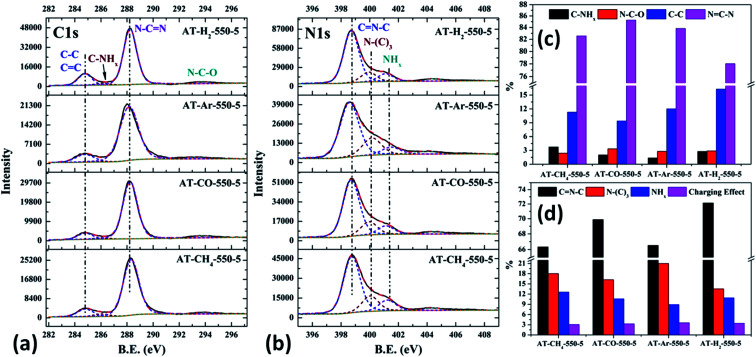
The high-resolution XPS spectra of C 1s (a), N 1s (b) and proportion of each state of C (c) and N (d) element for samples prepared in different atmospheres.

**Fig. 24:**
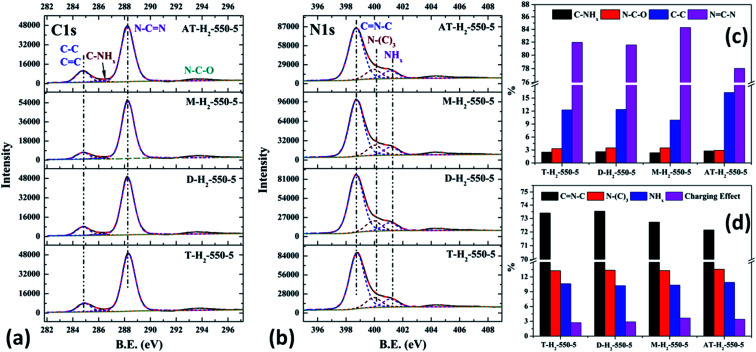
The high-resolution XPS spectra of C 1s (a), N 1s (b) and proportion of each state of C (c) and N (d) element for samples prepared by different precursors.

The XPS measurements were used to determine the elemental compositions and chemical states of the materials produced. Fig. 19 and 20 exhibit the survey spectrum for samples prepared at different conditions. Signals of the elements C, N, and O are displayed, but no peak assigned to S 2p_1/2_ (165 eV)^29,51^ can be seen in the spectrum survey. Higher resolution spectra were taken on the C 1s and N 1s regions as shown in Fig. 21–24. It can be seen from the figures that they all contain C 1s and N 1s signal peaks with similar binding energies, indicating that the C and N elements contained in the samples have the same chemical state. The O 1s spectra showed one main peak of the binding energy around 532 eV, which could be assigned to –OH groups on the surface of samples, which may be due to H_2_O or CO_2_ molecules adsorbed on the surface of the sample.^26,50^

As shown in Fig. 21(a), the high resolution spectrum of C 1s can be fitted into four peaks, indicating the presence of carbon elements in four different environments. The peak with binding energy of 284.8 eV is attributed to a special sp2 C–C bond in the CN from graphitic carbon or adsorbed amorphous carbon for peak position calibration. The peak at 286.2 eV belongs to the C atom on the aromatic ring connected to –NH_*x*_. The strong peak at 288.3 eV can be assigned to the sp^2^ hybrid C atom connected to N in the triazine ring N–C=N. The peak near 293.3 eV is derived from possible N–C–O.^52–54^ The backbone of g-C_3_N_4_ is bound by a heptazine ring with a terminal NH/NH_2_, which indicates that there are three types of N atoms in g-C_3_N_4_. The peak fitting results of the N 1s high-resolution map (such as Fig. 21(b)) show that the N atom binding energy of 398.6 eV can be ascribed to the sp^2^ hybrid nitrogen orbital in the triazine ring (C=N–C). Nitrogen with binding energy of 399.1 eV corresponds to bridging nitrogen atoms, that is, the sp^2^ hybrid nitrogen binds with three C atoms (N–(C)_3_).^25,55^ The peak observed at 401 eV is assigned to residual amino groups (N–H_2_) or sp^3^ hybrid nitrogens of the amino groups (C–N–H). In addition, the weak peak of N1s in the vicinity of 404.5 eV can be attributed to the localization of positive charges or the effect of charges on π excitation in heterocycles.^56,57^

The histogram of the proportions of different forms of C and N in the products obtained at different final pyrolysis temperatures were shown as Fig. 21(c) and (d). As AT–H_2_-400-5 contains a large amount of impurities, so it is not compared with other samples here. In the order of AT–H_2_-450-5, AT–H_2_-500-5 and AT–H_2_-550-5, the proportion of C existing as N=CN gradually decreases, which are 82.39%, 81.77%, and 78.02%, respectively; the proportion as C–NH_*x*_ is 3.26%, 3.01%, and 2.79%; the proportion as C–C attributable to graphitic carbon or amorphous carbon is 11.26%, 11.88%, and 16.29%. Among different forms of N, the proportion of N–(C)_3_ gradually decreases, which are 16.64%, 15.80%, and 13.54%, respectively; the proportion of C=N–C is 69.21%, 70.10%, and 72.14%, respectively. As the temperature increases, the material may undergo graphitization under the influence of H_2_ and high temperature. At the same time, the bridge N atom transformed to C=N–C, which makes the degree of polymerization of the material increase. In addition, the decrease of the proportion of N=C–N and the increase of the proportion of C=N–C in triazine indicate the generation of C defects in the material.

As shown in the Fig. 22(c) and (d), the proportion of C–NH_*x*_ slightly increases with the extension of the constant temperature time, and the proportion of N=C–N generally decreases. The proportion of NH_2_ of the N element in the obtained sample is almost the same, while the proportions of C=N–C and N–(C)_3_ are complementary. According to the analysis, due to the extension of the constant temperature time, the corrosion effect of H_2_ on the sample becomes more and more obvious. This is also a possible cause of the C defect, which is mainly reflected by the increase of the amorphous C content and the decrease of the N=C–N content.

As shown in Fig. 23(c) and 12(d), the distribution ratios of C and N elements in the products obtained under the atmospheres of CH_4_, CO, Ar, and H_2_ are as follows: N=CN accounts for 82.57%, 85.27%, 83.83%, and 78.02%; C=NC accounts for 66.33%, 69.89%, 66.51%, and 72.14%; N–(C)_3_ accounts for 18.03%, 16.24%, 21.01% and 13.54%, respectively. Among the products prepared under four different atmospheres, AT–H_2_-550-5 has the lowest ratio of N=CN and N–(C)_3_ and the highest ratio of C=NC, which indicate that the H_2_ atmosphere is more conducive to the transition from N–(C)_3_ to C=NC and the formation of C defects in the ring.

Among the various forms of C and N in AT–Ar-550-5, N–(C)_3_ and N=CN account for a relatively high proportion, while C=NC account for a relatively low proportion, and both the proportion of C–NH_*x*_ and NH_*x*_ were the lowest in the samples obtained in four different atmospheres, which indicate that the sheet structure of AT–Ar-550-5 was relatively large and there were obvious intra-ring nitrogen vacancies. In comparison with AT–Ar-550-5, the proportions of C=N–C in AT–CH_4_-550-5 has not changed significantly; N=CN and N–(C)_3_ ratios have decreased significantly, while the ratio of C–NH_*x*_ and NH_*x*_ increased, which indicates that CH_4_ will convert more N–(C)_3_ into NH_*x*_, and then produce more terminal carbon and terminal amino groups, causing fragmentation of the sheet structure. This is consistent with the shift of the (100) peak of AT–CH_4_-550-5 to a large diffraction angle in XRD patterns described above. Compared with the products made in the other three atmospheres (CH_4_, Ar, and H_2_), the largest N=CN ratio, the second highest C=NC ratio, and the smaller N–(C)_3_ ratio indicate that AT–CO-550-5 has a relatively complete ring structure and a large sheet structure.

As shown in the Fig. 24(c) and (d), the proportion of element C in the form of N=CN for the products prepared by using thiourea, dicyandiamide, melamine, and ammonium thiocyanate as precursors is 81.95%, 81.56%, 84.28%, and 78.02%, respectively; the proportion of C–NH_*x*_ is 2.50%, 2.60%, 2.34%, and 2.79%; the proportion of C–C is 12.25%, 12.36%, 9.91%, and 16.29%, respectively. In the N element composition of the above samples, the proportion of C=NC reaches 73.40%, 73.54%, 72.72%, and 72.14%; the proportion of N–(C)_3_ is 13.26%, 13.35%, 13.28%, and 13.54%; NH_*x*_ accounted for 10.62%, 10.22%, 10.33%, and 10.90%. The main differences are reflected in the relative content of N=C–N, C–C and C=N–C, which can be attributed to the differences in the paths of the precursors thermally polymerized to g-C_3_N_4_.

#### 3.1.7 UV-vis diffuse reflectance spectroscopy results

**Fig. 25:**
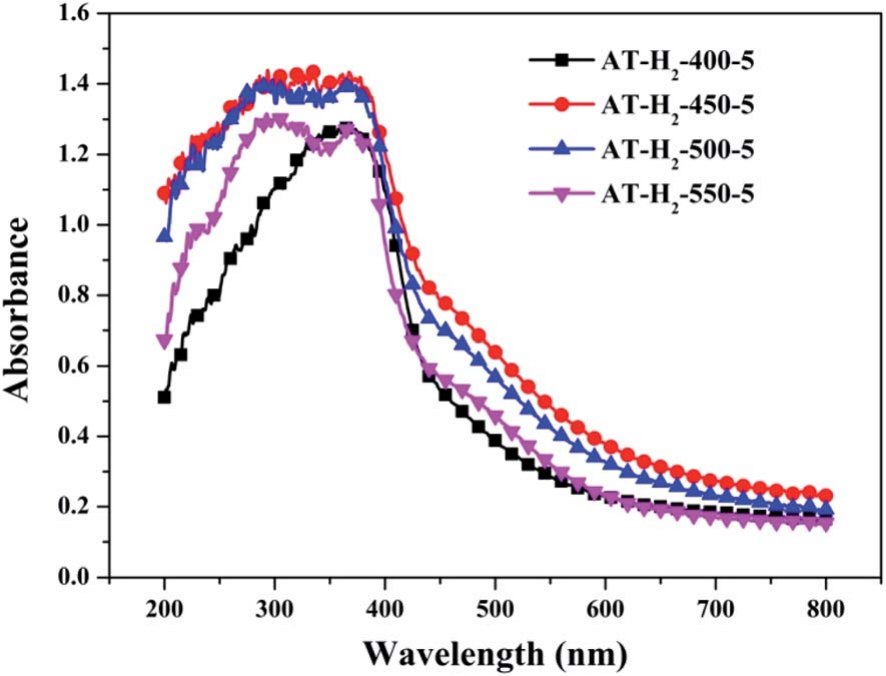
DSR UV-Vis diffuse reflectance spectra of samples prepared at different temperature.

**Fig. 26:**
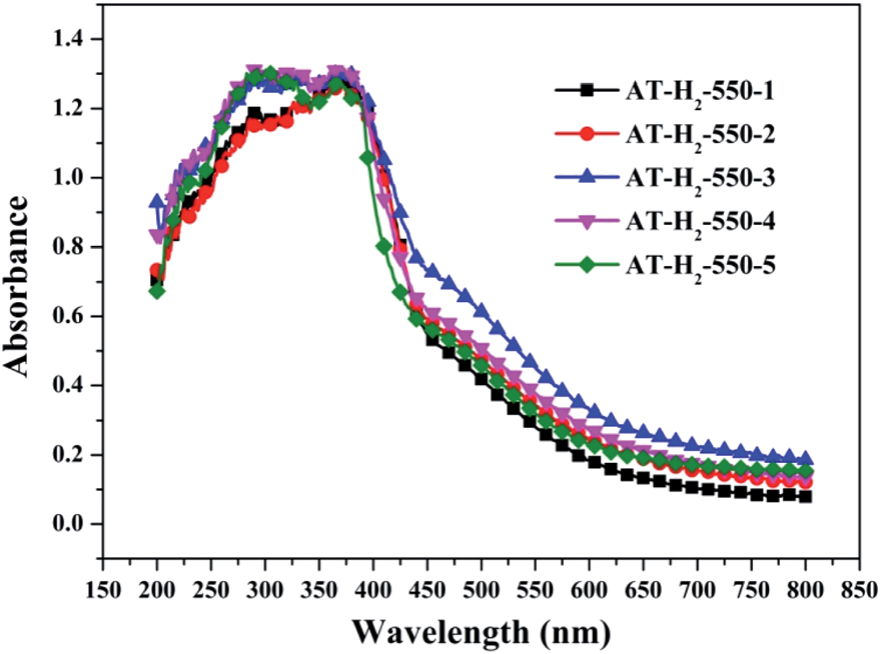
DSR UV-Vis diffuse reflectance spectra of the samples prepared in different temperature maintenance time.

**Fig. 27:**
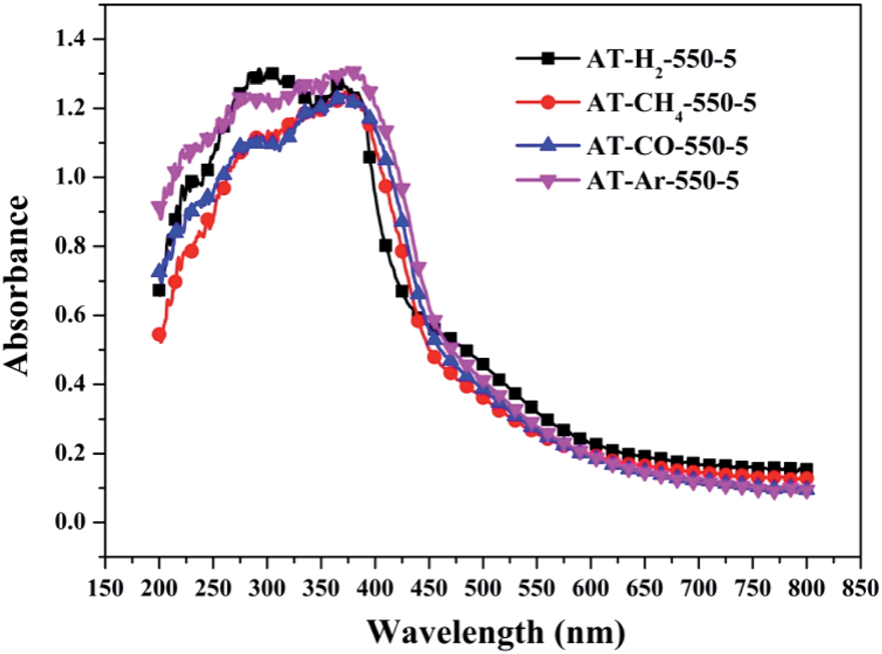
DSR UV-Vis diffuse reflectance spectra of the samples prepared in different atmospheres.

**Fig. 28:**
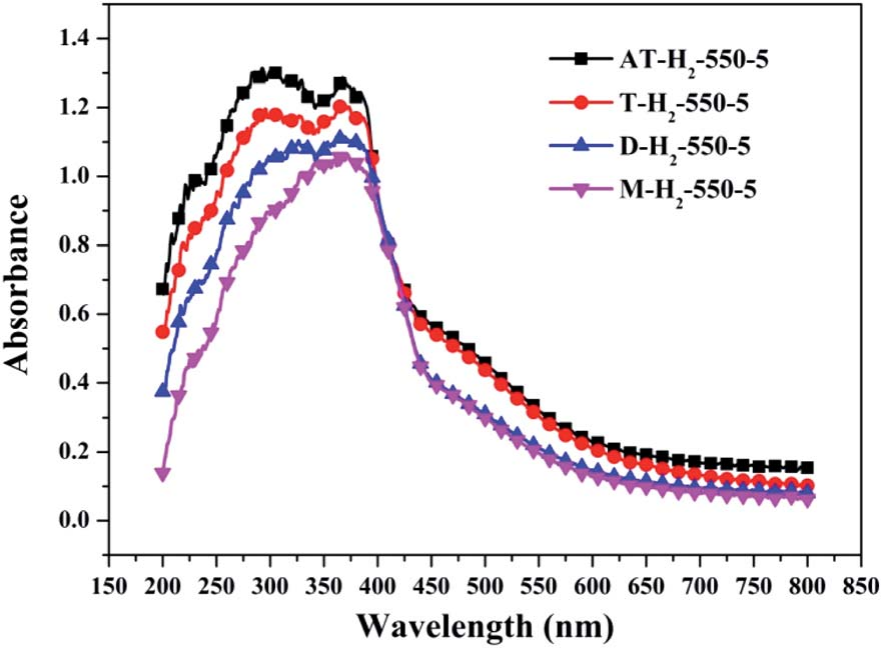
DSR UV-Vis diffuse reflectance spectra of the samples prepared by different precursors.

**Fig. 29:**
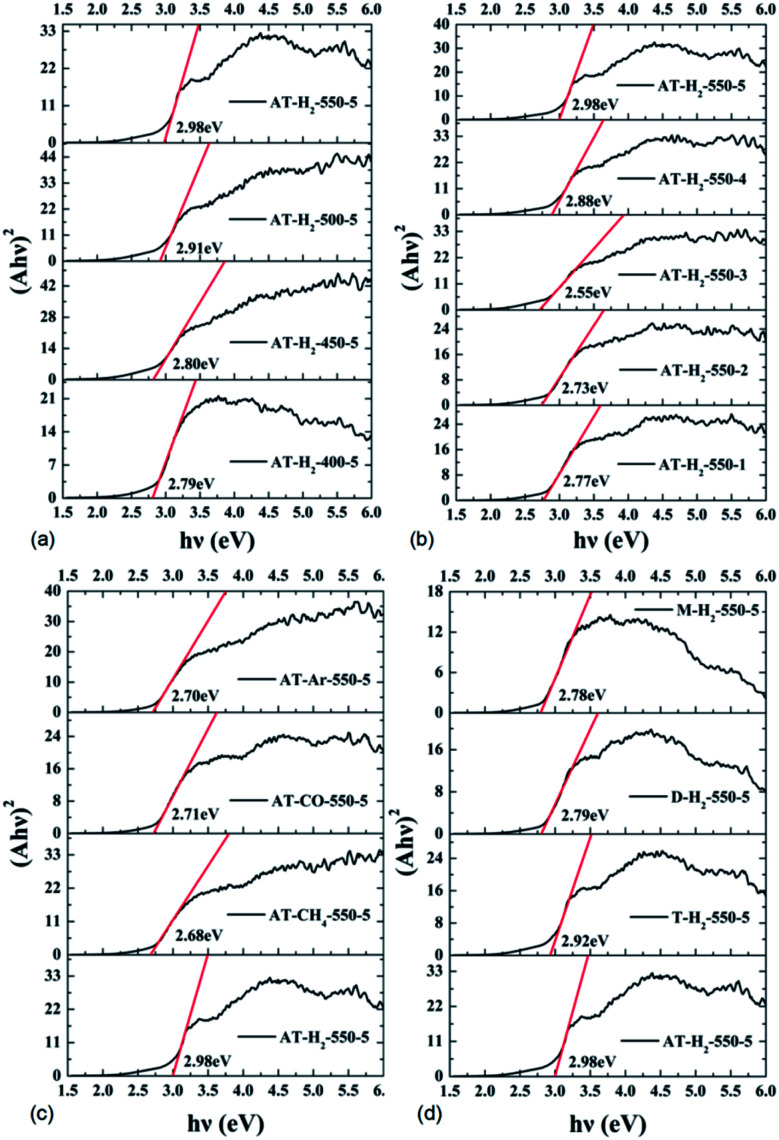
Tauc's plot of the prepared samples.

To determine the light absorption properties of the prepared materials, UV-Vis diffuse reflectance spectra were applied, and the data are plotted in Fig. 25–28. The band gap energies of the products could be calculated by plotting (*Ahν*)^2^*versus* the photon energy (*hν*) (Fig. 29) based on the Kubelka–Munk function.^42^

The band gap (*E*_CB_) and valence band (*E*_VB_) positions of the composite material can be calculated by the following formulas:^58–60^

2 *E*_CB_ = *X* − *E*_C_ − 1/2*E*_g_

**Fig. 30:**
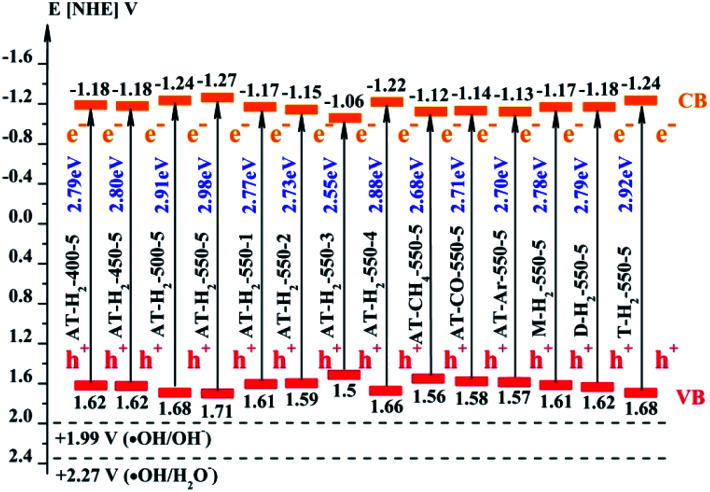
Schematic illustration of the band gap structure of the prepared samples.

3 *E*_VB_ = *E*_CB_ + *E*_g_

where *E*_VB_ and *E*_CB_ represent the valence band and conduction band position of the semiconductor material, *E*_C_ is the hydrogen electron free energy (4.5 eV), and *X* represents the electronegativity of the semiconductor material, wherein the *X* value of g-C_3_N_4_ is 4.72 eV.^61,62^ The positions of the conduction band and the valence band of RhB^63^ and as prepared g-C_3_N_4_ by the calculation are shown in Fig. 30.

Fig. 25 shows that g-C_3_N_4_ prepared under different conditions can respond to visible light. As seen from Fig. 25, as the pyrolysis temperature increases from 400 °C to 550 °C, the band gap of the product obtained by pyrolysis of ammonium thiocyanate in H_2_ atmosphere gradually increases, and the absorption spectrum of the sample shows a blueshift phenomenon, which is attributed to the quantum confinement effect of nanomaterials.^51^ As the constant temperature time increases from 1 h to 5 h, the band gap of the resulting products exhibits a decrease and then an increase. The band gap of the product obtained at a constant temperature time of 3 h was the smallest (2.55 eV), and the forbidden band width of the product at a constant temperature of 5 h was 2.98 eV. The band gaps of the products obtained under Ar, CO and CH_4_ atmospheres are basically the same, and all of them are approximately 2.70 eV, which is much smaller than the band gap of the product obtained in the H_2_ atmosphere, which matches the experimental results of photocatalytic evaluation (Fig. 35). In addition, the absorption properties of the products obtained by the thermal reaction of dicyandiamide with melamine are essentially the same; the products obtained by pyrolysis of thiourea and ammonium thiocyanate have similar band gaps; that is, their light absorption properties are also essentially consistent. The schematic illustration of the band gap structure of the prepared samples is shown as Fig. 30.

#### 3.1.8 Photoluminescence spectroscopy

**Fig. 31:**
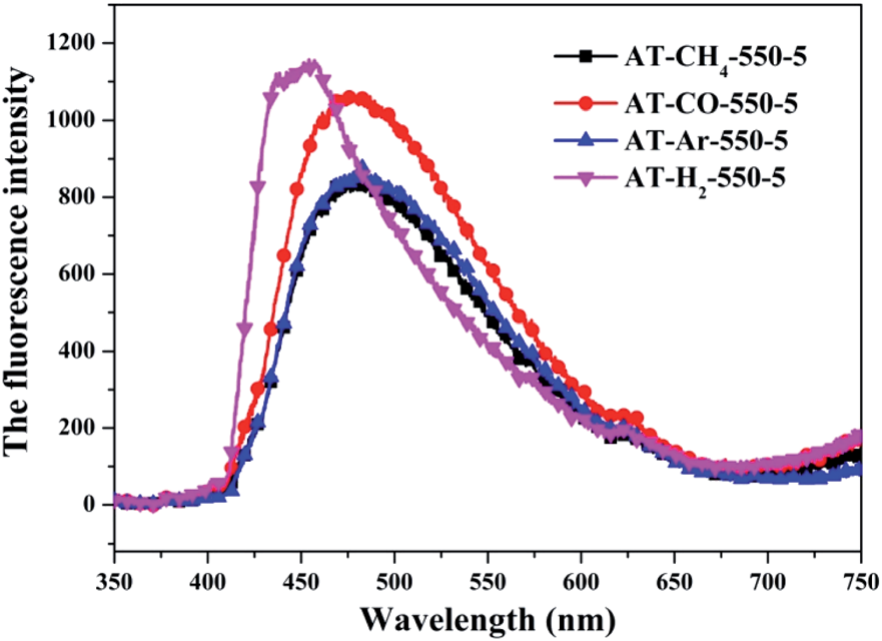
Photoluminescence spectra of samples prepared in different atmospheres.

**Fig. 32:**
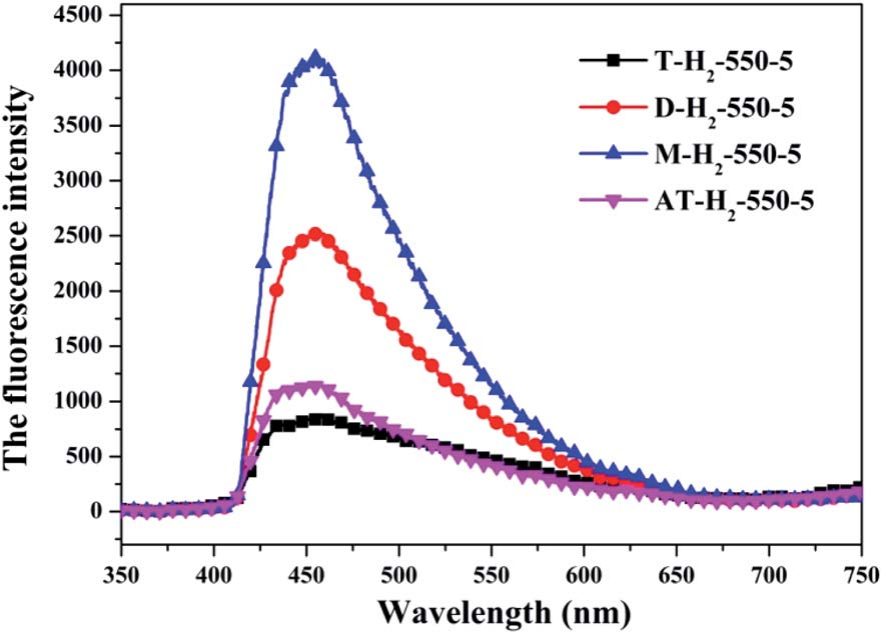
Photoluminescence spectra of all samples prepared in different atmospheres (a) and prepared by different precursors (b).

The photo-luminescence (PL) spectroscopy technique can be regarded as an important method to comprehend the transfer and recombination of the photo-induced electron–hole in a composite. Thus, the fluorescence emission spectra of carbon nitride prepared by different pyrolysis atmospheres and different precursors have been investigated as shown in Fig. 31 and 32, respectively. The wavelength of the excitation light source used is 420 nm. Fig. 31 shows that the fluorescence emission spectrum of the product obtained in the H_2_ atmosphere has a significant blue shift compared with the fluorescence emission spectrum of the product obtained in the Ar, CO and CH_4_ atmosphere, which is consistent with the widest band gap of AT–H_2_-550-5 in the DRS results. It is generally believed that the lower the intensity of the fluorescence spectrum of the sample, the stronger the ability of photo-generated carrier separation and transfer, the higher the photocatalytic activity.

The PL spectrum of the product obtained by thermal polymerization of four precursors in an H_2_ atmosphere is shown in Fig. 31. The PL spectrum of AT–H_2_-550-5 and T–H_2_-550-5 displayed a platform emission peak between 437–456 nm due to the band-to-band recombination of electron–hole. The PL peak intensities are sequentially increased in order of T–H_2_-550-5 < AT–H_2_-550-5 < D–H_2_-550-5 < M–H_2_-550-5, demonstrating that the recombination of photo-generated electron–hole pairs was more suppressed for AT–H_2_-550-5 and T–H_2_-550-5 than that for D–H_2_-550-5 and M–H_2_-550-5, which is advantageous for explaining the relationship between the performance of each catalyst shown in Fig. 36 for the photocatalytic degradation of RhB. In addition, compared to M and D, the PL spectra of AT and T are slightly blue-shifted, which can also correspond well to the DRS results. In general, this happens in the presence of a suitable π–π conjugated electronic system, which was found to be beneficial for the migration of photo-generated electrons, thereupon restricting the recombination of photo-produced electron–hole.^53^

It should be noted that T–H_2_-550-5 exhibits the lowest PL intensity, but its photocatalytic RhB performance is still inferior to AT–H_2_-550-5. It can be seen that the recombination of photogenerated charges is not the determinant of the photocatalyst's catalytic activity here. The reasons that influence the activity of the materials in this paper may be attributed more to the increased reactive sites of the large specific surface area and the enhanced redox capacity due to the wide band gap.

### 3.2 Photocatalytic activities of samples

#### 3.2.1 Photocatalytic degradation of RhB

**Fig. 33:**
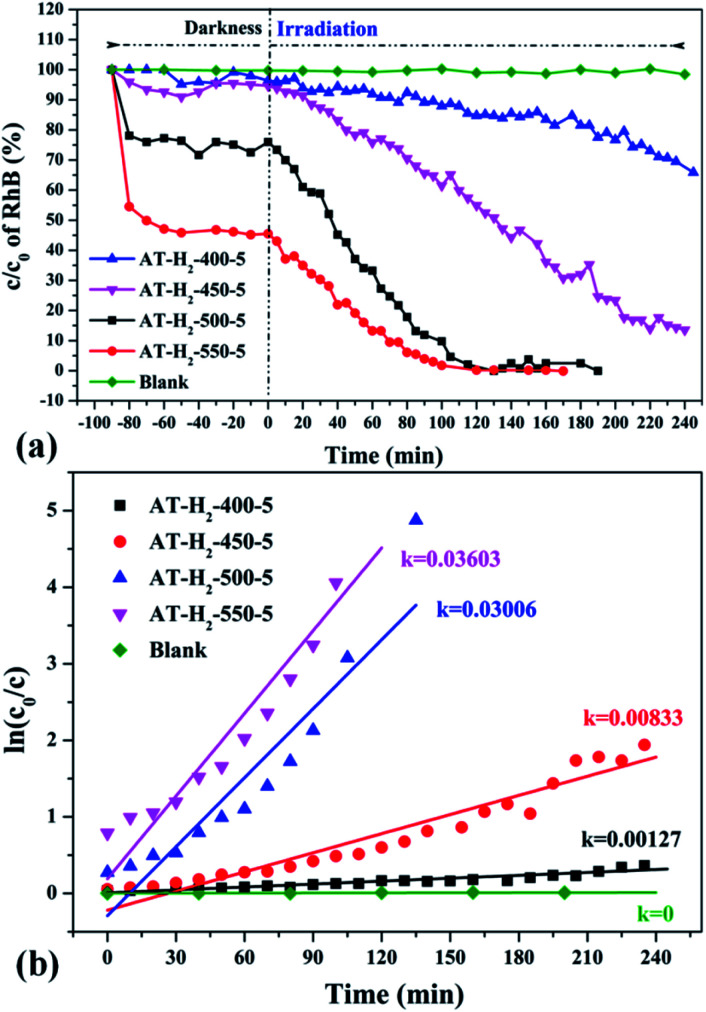
(a) Photo degradation of RhB over the synthesized g-C_3_N_4_ at different temperature (b) influence of photocatalyst preparation temperature on degradation kinetics.

**Fig. 34:**
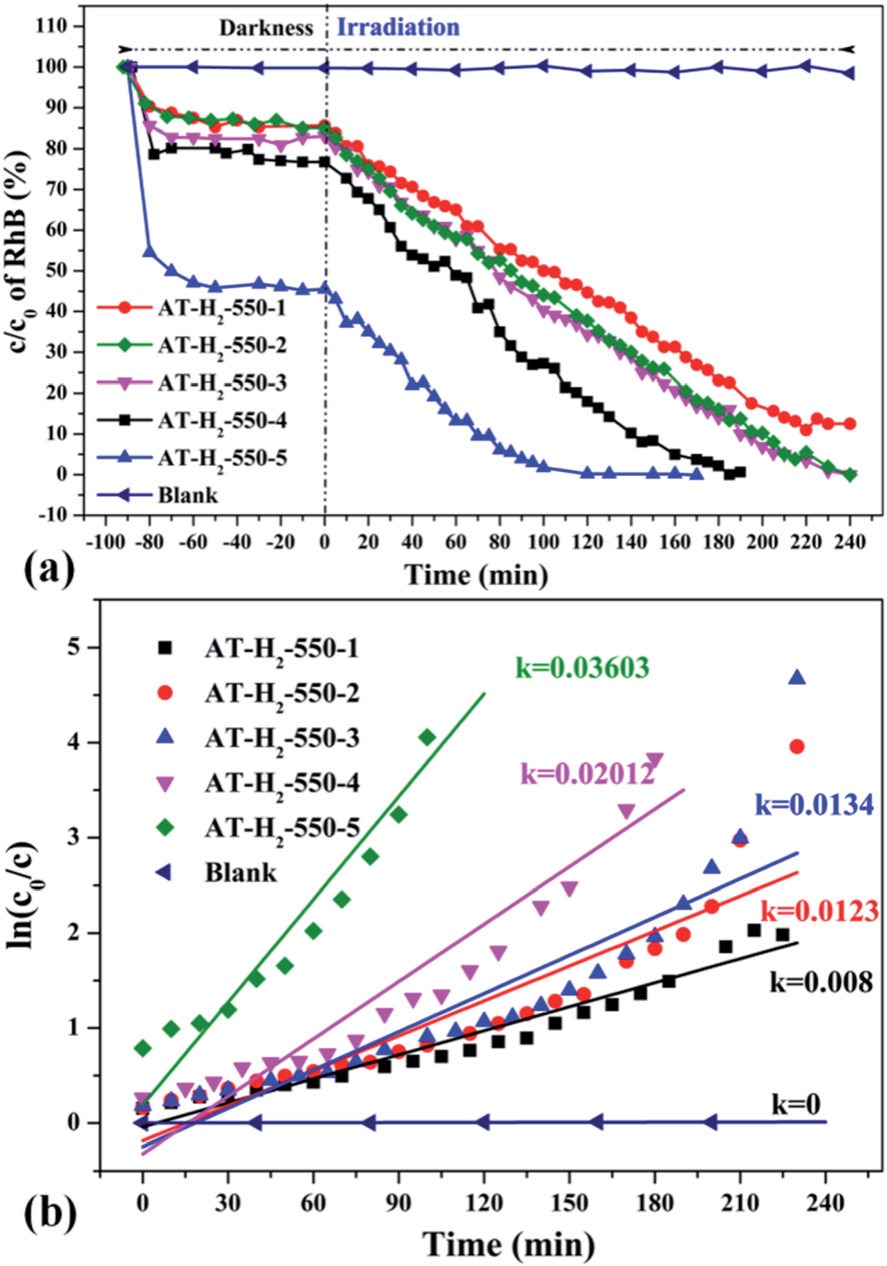
The degradation curve of RhB (a) photocatalyzed by g-C_3_N_4_ prepared in different temperature maintenance time and the first-order kinetic fitting curve(b).

**Fig. 35:**
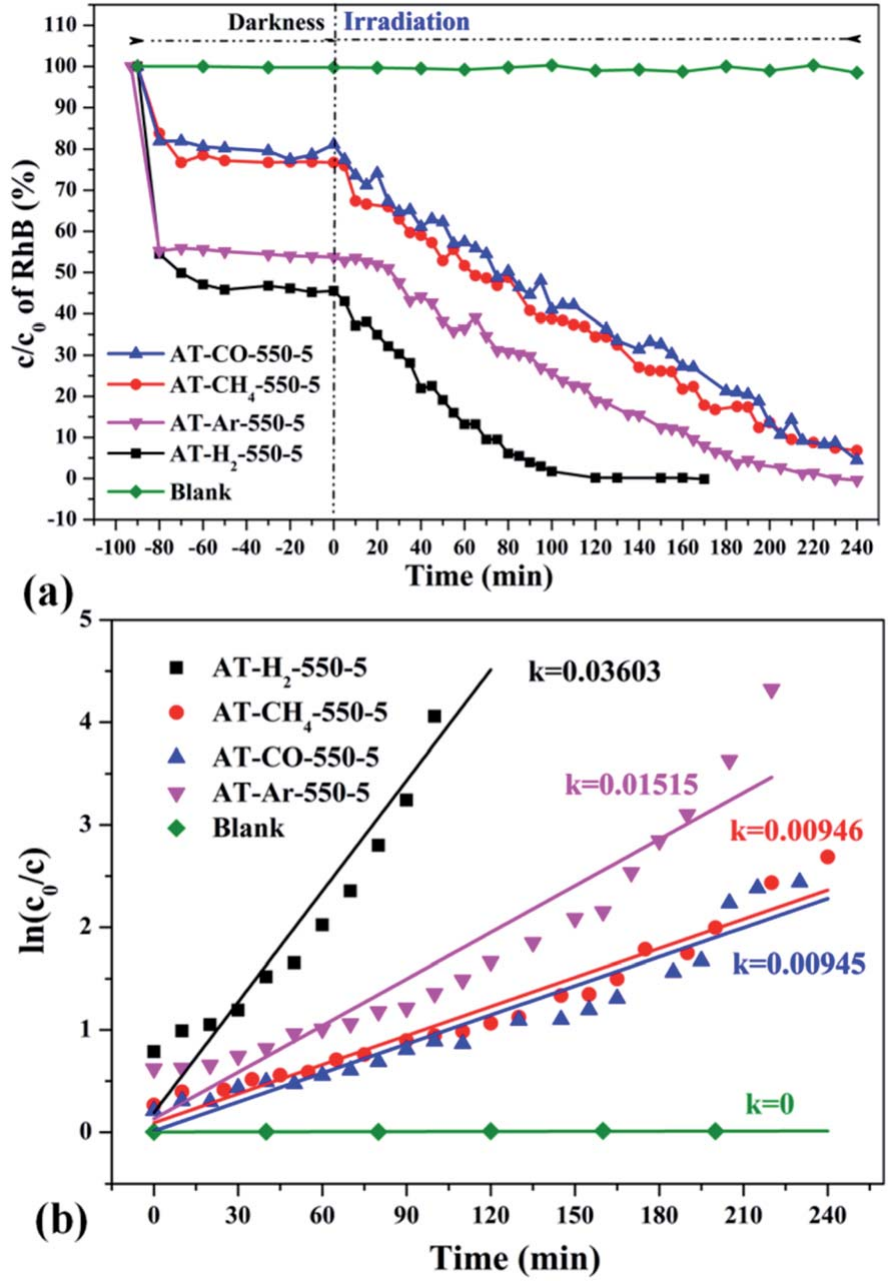
The degradation curve of RhB (a) photocatalyzed by g-C_3_N_4_ synthesized in different atmospheres and their first-order kinetic fitting curve (b).

**Fig. 36:**
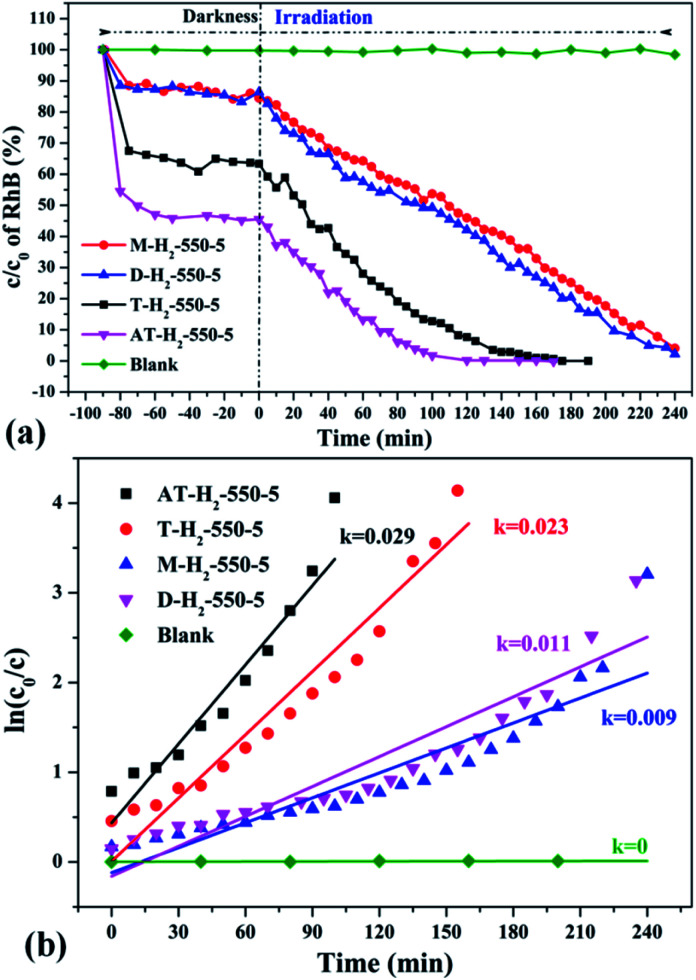
The degradation curve of RhB (a) photocatalyzed by g-C_3_N_4_ synthesized by different precursors and the first-order kinetic fitting curve (b).

To eliminate the influence of the degradation of RhB in the absence of photocatalyst, the degradation experiment of RhB without photocatalyst was also carried out, and the experimental data showed that the RhB is quite stable. Fig. 33–36 show the activity evaluation curve of the photocatalytic degradation of RhB solution obtained by g-C_3_N_4_ under different preparation conditions. As shown in Fig. 33(a), as the pyrolysis temperature is gradually increased from 400 °C to 550 °C, the adsorption characteristics of the obtained catalysts are gradually increased, being 3%, 5%, 25% and 56%, respectively. After 4 h of blue light illumination, the photocatalytic RhB degradation rates of the pyrolysis products at 400 °C and 450 °C were 34% and 87%, respectively. The pyrolysis products at 500 °C and 550 °C can catalyze the complete degradation of RhB after 2 h of illumination. Moreover, the first-order constant (*k*) for photocatalytic RhB degradation of g-C_3_N_4_ prepared at different temperature was calculated by ln(*C*_0_/*C*) = *kt*. The time-course variation of ln(*C*_0_/*C*) and the kinetic constants of the samples were shown in Fig. 33(b). The result showed that as the final pyrolysis temperature rises, the pyrolysis kinetic constant also gradually increases.

It can be seen from Fig. 34(a) that as the constant temperature time increases from 1 h to 5 h at 550 °C, the adsorption capacity of the obtained catalyst also gradually increases. When the dark adsorption equilibrium was reached, the adsorption rates of the respective catalysts for the RhB solution were 16%, 16%, 17%, 24%, and 56%. This is attributed to the fact that the polymerization degree of the material is gradually increased with the extension of the constant temperature time and that the specific surface area, pore volume and pore structure of the sample are increased, which is favorable for the adsorption of the degraded material. After 4 hours of light irradiation, the degradation rate of AT–H_2_-550-1-catalyzed RhB was 88%, and the degradation rates of RhB by AT–H_2_-550-2 and AT–H_2_-550-3 were almost 100%.

Fig. 35 shows the activity evaluation curve of the photocatalytic degradation of RhB by pyrolysis products of NH_4_SCN as precursor in H_2_, CH_4_, CO and Ar atmospheres. It can be seen that CO and CH_4_ have similar effects on the photocatalytic activity of NH_4_SCN pyrolysis products. The adsorption capacity of RhB in dark treatment was 22%, and the percentage of degradation of RhB after irradiation for 4 hours reached 96%. The adsorption capacity of AT–Ar-550-5 (adsorption rate of 47%) and photocatalytic activity (RhB degradation rate of almost 100% after 4 hours of illumination) are superior to those of AT–CO-550-5 and AT–CH_4_-550-5, but not as good as in the case of AT–H_2_-550-5. This can be more intuitively reflected by the pyrolysis kinetic constants in Fig. 35(b).

Fig. 36 exhibits evaluation curves of the photocatalytic degradation of RhB by g-C_3_N_4_ prepared by different kind of precursors in H_2_ atmosphere at 550 °C for 5 h. It is obvious that the products prepared by using dicyandiamide and melamine as precursors have similar adsorption characteristics (14%) and photocatalytic degradation of RhB (98% after 4 hours of illumination). The adsorption performance (37%) and photocatalytic performance (approximately 100% of RhB degradation ability after 3 hours of illumination) of the product obtained with thiourea as the precursor are superior to those of the above two precursors. The kinetic constant of the above reaction shows that the AT–H_2_-550-5 exhibits nearly three times the photocatalytic capacity of M–H_2_-550-5.

#### 3.2.2 Catalyst stability test results

The stability and reusability of catalysts were of significant importance for practical application. Therefore, we tested four successive cycles for the photocatalytic degradation of RhB by another batch of AT–H_2_-500-5.

**Fig. 37:**
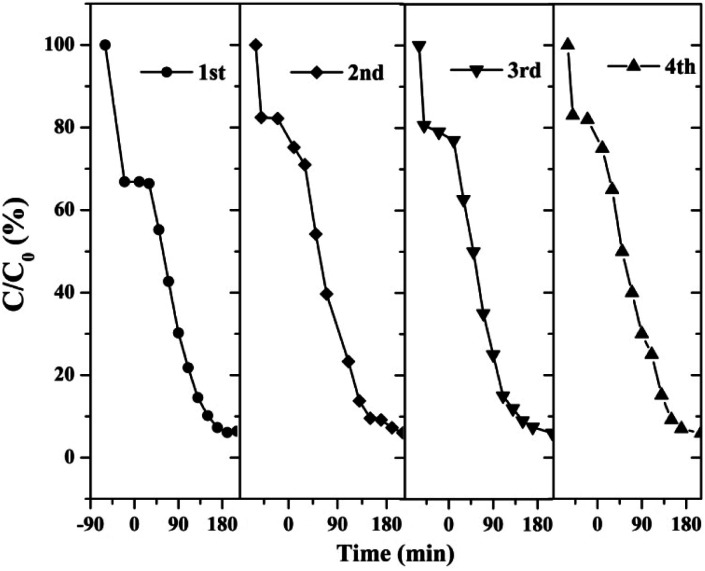
Cyclic stability test of photo-catalytic degradation of RhB by prepared samples.

Fig. 37 shows that the as-prepared material has good recyclability. In addition to the decrease in adsorption performance compared to the initial sample, there is no significant deactivation of the catalyst after 4 cycles in RhB degradation. The cycling experiments determined the stability and reusability of the samples.

### 3.3 Free radical capture experiment results

**Fig. 38:**
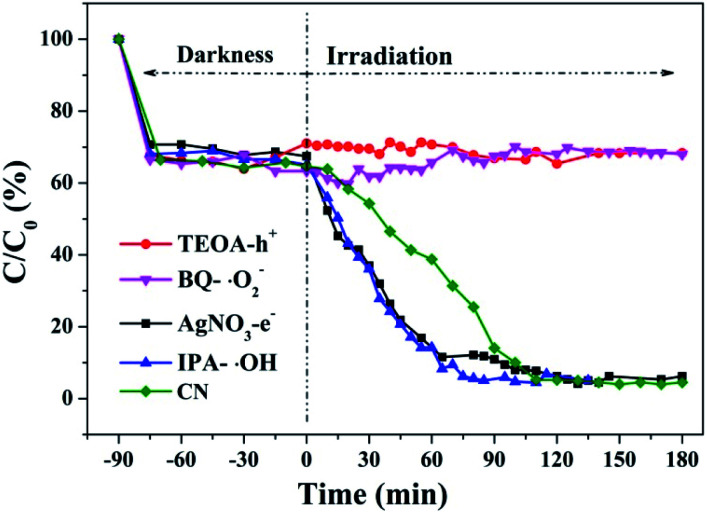
Experimental results of free radical capture during photo catalytic degradation of RhB by the prepared samples.

Hydroxyl radicals (˙OH), holes (h^+^) and superoxide radicals (˙O^2−^) are considered to be common active substances in the degradation of photocatalytic organic compounds. Therefore, in this study, different sacrificial agents were added to the reaction system to determine which radicals act in the photocatalytic reaction in order to determine the reaction mechanism. Next, 1 mM TEOA, BQ, IPA and AgNO_3_ were added to the reaction system as hole (h^+^), superoxide radical (˙O^2−^), hydroxyl radical (˙OH) and electron (e^−^) trapping agents, respectively. The RhB degradation curves (Fig. 38) obtained show that after TEOA and BQ were added into the reaction system, RhB almost did not degrade after dark adsorption. When IPA and AgNO_3_ were added into the reaction system, the catalytic effect of g-C_3_N_4_ on RhB degradation was enhanced compared with that of g-C_3_N_4_ alone. When electron trapping agent is added to the reaction system, the recombination of photogenerated carriers is weakened and the reaction process is accelerated. It can be concluded that the main active components of photocatalytic RhB degradation should be holes (h^+^) and superoxide radicals (˙O^2−^).

Based on the above results and discussion, the reaction mechanism of the photocatalytic degradation of RhB by g-C_3_N_4_ under blue light irradiation was proposed.

As the CB position of as prepared g-C_3_N_4_ (−1.06 ∼ −1.27 eV) are more negative than that of O_2_/˙O_2_^−^ (−0.33 eV *vs.* NHE),^64^ the photogenerated electrons of samples could easily reduce the oxygen molecules absorbed on the samples or dissolved in solution to produce the superoxide radical (˙O_2_^−^) and hydrogen peroxide (H_2_O_2_),^65–67^ which can further create the hydroxyl radical (˙OH). The resulting oxygen-active species will oxidize the RhB into CO_2_ and H_2_O. In addition, the VB positions of samples (+1.5 ∼ +1.71 eV) are more negative than those of ˙OH/OH^−^ (+1.99 eV *vs.* NHE)^68,69^ and ˙OH/H_2_O (+2.27 eV *vs.* NHE), which means the photo excited holes on the VB of samples had no ability to oxidize the H_2_O molecules or hydroxyl (OH^−^) to hydroxyl radical (˙OH),^64^ but can directly oxidize RhB to decompose. The above results are consistent with the results of the free radical trapping experiment: that is, OH is not the main active substance in the photodegradation of RhB. The photodegradation of RhB is achieved by the synergistic catalysis of ˙O^2−^ and h^+^.

4 g-C_3_N_4_ + *hν* → e_CB_^−^ + *h*_VB_^+^

5 O_2_ + e^−^ → ˙O_2_^−^/H_2_O_2_ → ˙OH

6 ˙O_2_^−^/˙OH/h^+^ + RhB → CO_2_ + H_2_O

## 4 Conclusions

When NH_4_SCN is used as a precursor to prepare g-C_3_N_4_, the increase of thermal polymerization temperature (400–550 °C) and the extension of constant temperature time (1–5 h) are beneficial to increase the specific surface area of the obtained sample, reduce the interlayer spacing, and enhance the crystallinity of the sample. At the same time, the amount of RhB adsorbed by the material and the photocatalytic degradation ability gradually increased. The effect of hydrogen bonding in the material structure makes the interlayer spacing of products obtained in H_2_ and CH_4_ atmospheres smaller than those prepared in Ar and CO. The band gaps of the products obtained in Ar, CO, and CH_4_ are basically the same, all about 2.70 eV, which are much smaller than that obtained in H_2_. The H_2_ atmosphere is more conducive to the transition of bridging N atoms to N in the triazine ring and the formation of C defects in the ring. The Ar atmosphere product has more obvious nitrogen vacancy in the ring. CH_4_ can promote the conversion of more bridged nitrogen to NH_*x*_ to produce more terminal carbons and terminal amino groups, which in turn causes the layered structure to break. The adsorption capacity and photocatalytic activity of the products obtained in Ar and H_2_ atmospheres are better than those produced in CO and CH_4_, which can be attributed to the combined effect of large specific surface area and structural defects of the materials. The g-C_3_N_4_ prepared with AT, T, D and M as precursors has a similar sheet-like stacking structure and a clear UV-visible light absorption capability. However, the sample's large specific surface area, wide band gap, and excellent photogenerated carrier separation and transfer capabilities make the adsorption performance and photocatalytic performance of the products obtained with AT and T as precursors better than those prepared with D and M as precursors.

This study has important theoretical significance and application background for the treatment of desulfurization waste liquid with waste heat of reducing waste gas and the preparation of g-C_3_N_4_ catalyst using the main salt of the desulfurization waste liquid as raw materials, which opens up a new direction for the treatment of the main salt of the desulfurization waste liquid.

## Conflicts of interest

There are no conflicts to declare.

## Supplementary Material

RA-010-D0RA02454F-s001

## References

[cit1] Xiao M., Luo B., Wang S., Wang L. (2018). J. Energy Chem..

[cit2] Jiang T., Du Y., Dong M., Zhao Q. (2019). New J. Chem..

[cit3] Liu G., Niu P., Sun C. H., Smith S. C., Chen Z. G., Lu G. Q., Cheng H. M. (2010). J. Am. Chem. Soc..

[cit4] Kang Y., Yang Y., Yin L.-C., Kang X., Wang L., Liu G., Cheng H.-M. (2016). Adv. Mater..

[cit5] Zhou Z., Zhang Y., Shen Y., Liu S., Zhang Y. (2018). Chem. Soc. Rev..

[cit6] Liao G., Gong Y., Zhang L., Gao H., Yang G.-J., Fang B. (2019). Energy Environ. Sci..

[cit7] Liu A. Y., Cohen M. L. (1989). Science.

[cit8] Liu A. Y., Wentzcovitch R. M. (1994). Phys. Rev. B: Condens. Matter Mater. Phys..

[cit9] Lowther J. E. (1999). Phys. Rev. B: Condens. Matter Mater. Phys..

[cit10] Ortega J., Sankey O. F. (1995). Phys. Rev. B: Condens. Matter Mater. Phys..

[cit11] Wang X., Maeda K., Thomas A., Takanabe K., Xin G., Carlsson J. M., Domen K., Antonietti M. (2009). Nat. Mater..

[cit12] Wang X., Maeda K., Chen X., Takanabe K., Domen K., Hou Y., Fu X., Antonietti M. (2009). J. Am. Chem. Soc..

[cit13] Wang X., Maeda K., Thomas A., Takanabe K., Xin G., Carlsson J. M., Domen K., Antonietti M. (2009). Nat. Mater..

[cit14] Sheng C., Wang C., Feng J., Ying W., Zou Z. (2014). Int. J. Hydrogen Energy.

[cit15] Yong W., Xinchen W., Markus A. (2012). Angew. Chem..

[cit16] Katharina S., Mesch M. B., Viola D., Christian Z., Jürgen S., Lotsch B. V. (2014). J. Am. Chem. Soc..

[cit17] Yuan X. , PhD thesis, YanShan University, 2018

[cit18] Komatsu T. (2001). J. Mater. Chem..

[cit19] Lotsch B. V., Wolfgang S. (2010). Chem.–Eur. J..

[cit20] Zhang Y., Pan Q., Chai G., Liang M., Dong G., Zhang Q., Qiu J. (2013). Sci. Rep..

[cit21] Fang H., Gang C., Yaoguang Y., Yansong Z., Yi Z., Sue H. (2015). Chem. Commun..

[cit22] Solozhenko V. L., Solozhenko E. G., Zinin P. V., Li C. M., Chen J., Parise J. B. (2003). J.
Phys. Chem. Solids.

[cit23] Zhao Y. C., Yu D. L., Zhou H. W., Tian Y. J., Yanagisawa O. (2005). J. Mater. Sci..

[cit24] Juncao B., Jianfu L., Sergii K., Yu W., Qian L., Tsz Chun L., Niehaus T. A., Rogach A. L., Rui-Qin Z. (2015). ChemPhysChem.

[cit25] Zhao Z., Fan J., Xue Y., Chang H., Masubuchi Y., Yin S. (2018). J. Alloys Compd..

[cit26] Gerardo A. S., Nikolai S., Chong S. Y., Torbj?Rn B. R., Palgrave R. G., Andrea L., Markus A., Khimyak Y. Z., Krasheninnikov A. V., Rabe J. P. (2014). Angew. Chem..

[cit27] Torres-Pinto A., Sampaio M. J., Silva C. G., Faria J. L., Silva A. M. T. (2019). Appl. Catal., B.

[cit28] Lima M. J., Silva A. M. T., Silva C. G., Faria J. L. (2017). J. Catal..

[cit29] Cui Y., Zhang J., Zhang G., Huang J., Liu P., Antonietti M., Wang X. (2011). J. Mater. Chem..

[cit30] Lotsch B. V., Döblinger M., Sehnert J., Seyfarth L., Senker J., Oeckler O., Schnick W. (2010). Chem.

[cit31] Fina F., Callear S. K., Carins G. M., Irvine J. T. S. (2015). Chem. Mater..

[cit32] Gillan E. G. (2000). Chem. Mater..

[cit33] Amorin L. H., Suzuki V. Y., de Paula N. H., Duarte J. L., Toledo da Silva M. A., Taft C. A., La Porta F. d. A. (2019). New J. Chem..

[cit34] Ismael M., Wu Y., Taffa D. H., Bottke P., Wark M. (2019). New J. Chem..

[cit35] Li K., Sun M., Zhang W.-D. (2018). Carbon.

[cit36] Hu C., Hung W.-Z., Wang M.-S., Lu P.-J. (2018). Carbon.

[cit37] Wu Y., Liu L.-M., An X., Wei T. (2019). New J. Chem..

[cit38] Liu H., Zhu X., Han R., Dai Y., Sun Y., Lin Y., Gao D., Wang X., Luo C. (2020). New J. Chem..

[cit39] Dongya Ni Y. Z., Shen Y., Liu S., Zhang Y. (2020). Chin. Chem. Lett..

[cit40] Ziyu Gan C. H., Shen Y., Zhou Q., Han D., Ma J., Liu S., Zhang Y. (2020). Chin. Chem. Lett..

[cit41] Zhao T., Zhou Q., Lv Y., Han D., Wu K., Zhao L., Shen Y., Liu S., Zhang Y. (2020). Angew. Chem., Int. Ed..

[cit42] Zhao H., Wang S., He F., Zhang J., Chen L., Dong P., Tai Z., Wang Y., Gao H., Zhao C. (2019). Carbon.

[cit43] Inagaki M., Tsumura T., Kinumoto T., Toyoda M. (2019). Carbon.

[cit44] Zhang S., Li G., Wang H., Li C., Li T., Zhang Y. (2018). J. Anal. Appl. Pyrolysis.

[cit45] Qitao Z. , PhD thesis, Yangzhou University, 2017

[cit46] Hong P. J., Xiwang Z., Alan Jianhong D., Sun D. D., Leckie J. O. (2008). J. Am. Chem. Soc..

[cit47] Hexing L., Zhenfeng B., Jian Z., Yuning H., Hui L., Yunfeng L. (2007). J. Am. Chem. Soc..

[cit48] Tian L., Li J., Liang F., Wang J., Li S., Zhang H., Zhang S. (2017). Appl. Catal., B.

[cit49] Dong H., Guo X., Yang C., Ouyang Z. (2018). Appl. Catal., B.

[cit50] Yu Y., Wang C., Luo L., Wang J., Meng J. (2018). Chem. Eng. J..

[cit51] Cao S., Fan B., Feng Y., Chen H., Jiang F., Wang X. (2018). Chem. Eng. J..

[cit52] Yang Z., Hu K., Meng X., Tao Q., Dong J., Liu B., Lu Q., Zhang H., Sundqvist B., Zhu P. (2017). Carbon.

[cit53] Song T., Zhang P., Wang T., Ali A., Zeng H. (2018). Appl. Catal., B.

[cit54] Wu J., Li N., Fang H.-B., Li X., Zheng Y.-Z., Tao X. (2019). Chem. Eng. J..

[cit55] Zhuang J., Zhang J., Pang J., Wang A., Wang X., Zhu W. (2019). Dyes Pigm..

[cit56] Zhang D., Guo Y., Zhao Z. (2018). Appl. Catal., B.

[cit57] Yu H., Shi R., Zhao Y., Bian T., Zhao Y., Zhou C., Waterhouse G. I. N., Wu L.-Z., Tung C.-H., Zhang T. (2017). Adv. Mater..

[cit58] Yaocheng D. , PhD thesis, Hunan University, 2018

[cit59] Li Y., Fang L., Jin R., Yang Y., Fang X., Xing Y., Song S. (2014). Nanoscale.

[cit60] Meng S., Ning X., Zhang T., Chen S. F., Fu X. (2015). Phys. Chem. Chem. Phys..

[cit61] Xu D., Bei C., Cao S., Yu J. (2015). Appl. Catal., B.

[cit62] Huang L., Hui X. U., Zhang R., Cheng X., Xia J., Yuanguo X. U., Huaming L. I. (2013). Appl. Surf. Sci..

[cit63] Mamba G., Mishra A. K. (2016). Appl. Catal., B.

[cit64] Wanjun W., Yu J. C., Dehua X., Po Keung W., Yecheng L. (2013). Environ. Sci. Technol..

[cit65] Lin B., Yang G., Yang B., Zhao Y. (2016). Appl. Catal., B.

[cit66] Xue C., Wang T., Yang G., Yang B., Ding S. (2014). J. Mater. Chem. A.

[cit67] Xiao S., Zhu W., Liu P., Liu F., Dai W., Zhang D., Chen W., Li H. (2016). Nanoscale.

[cit68] Liu Q., Guo Y., Chen Z., Zhang Z., Fang X. (2016). Appl. Catal., B.

[cit69] Cui Y., Ding Z., Liu P., Antonietti M., Fu X., Wang X. (2012). Phys. Chem. Chem. Phys..

