# Supplementary material for: g-C3N4 synthesized from NH4SCN in a H2 atmosphere as a high performance photocatalyst for blue light-driven degradation of rhodamine B
Source: RSC Adv. 2020 May 22;10(33):19669–85. doi: 10.1039/d0ra02454f (PMC9054138; doi:10.1039/d0ra02454f)
Supplement: RA-010-D0RA02454F-s001 [file RA-010-D0RA02454F-s001.pdf]

Electronic Supplementary Material (ESI) for RSC Advances.
This journal is © The Royal Society of Chemistry 2020

Table S1 The specific surface area (S_{BET}, m^2g^{-1}), total pore volume (V_{pore}, cm^3g^{-1}), and BJH average pore width (D_{pore}, nm) of the prepared samples

Sample	Specific surface area (S_{BET}, m^2g^{-1})	BJH Cumulative pore volume (V_{pore}, cm^3g^{-1})	BJH Average pore width (D_{pore}, nm)
AT-H_2-550-1	24.0479	0.1323	17.18
AT-H_2-550-2	26.7601	0.1557	17.60
AT-H_2-550-3	32.5350	0.2780	23.51
AT-H_2-550-4	40.3711	0.2870	20.97
AT-H_2-550-5	65.8849	0.3874	19.46
AT-H_2-400-5	3.0977	0.0224	18.36
AT-H_2-450-5	40.5149	0.3063	22.49
AT-H_2-500-5	54.1686	0.4566	24.19
AT-H_2-550-5	65.8849	0.3874	19.46
AT-CH_4-550-5	13.6830	0.1395	23.15
AT-CO-550-5	16.5323	0.1775	24.27
AT-Ar-550-5	20.5713	0.1293	18.45
AT-H_2-550-5	65.8849	0.3874	19.46
T-H_2-550-5	54.7424	0.3577	20.10
D-H_2-550-5	23.9363	0.1479	16.96
M-H_2-550-5	24.5017	0.1579	17.39
AT-H_2-550-5	65.8849	0.3874	19.46

Table S2 The C, H, N, S elemental analysis data and C/N molar ratio of the samples

Sample	m%				n%				(n/n)
	C	N	H	S	C	N	H	S	C/N
AT-H_2-400-5	32.070	60.400	2.319	1.286	2.670	4.312	2.301	0.040	0.619
AT-H_2-450-5	33.510	58.920	2.312	0	2.790	4.207	2.294	0.000	0.663
AT-H_2-500-5	34.175	59.370	2.387	0	2.845	4.239	2.368	0.000	0.671
AT-H_2-550-5	35.215	60.895	2.567	0	2.932	4.348	2.547	0.000	0.674
AT-H_2-550-1	35.050	61.420	2.030	0	2.918	4.385	2.014	0.000	0.665
AT-H_2-550-2	34.470	61.305	2.248	0	2.870	4.377	2.230	0.000	0.656
AT-H_2-550-3	34.680	61.345	2.018	0	2.887	4.380	2.002	0.000	0.659
AT-H_2-550-4	34.345	61.370	2.352	0	2.860	4.381	2.333	0.000	0.653
AT-H_2-550-5	35.215	60.895	2.567	0	2.932	4.348	2.547	0.000	0.674
AT- CH_4-550-5	34.545	61.295	1.649	0	2.876	4.376	1.636	0.000	0.657
AT- CO -550-5	34.530	61.480	2.245	0	2.875	4.389	2.227	0.000	0.655
AT- Ar -550-5	34.540	61.435	1.866	0	2.876	4.386	1.851	0.000	0.656
AT-H_2-550-5	35.215	60.895	2.567	0	2.932	4.348	2.547	0.000	0.674
D-H_2-550-5	35.045	62.615	1.571	0	2.918	4.470	1.559	0.000	0.653
M-H_2-550-5	35.190	62.860	1.955	0	2.930	4.488	1.940	0.000	0.653
T-H_2-550-5	35.730	62.095	2.158	0	2.975	4.433	2.141	0.000	0.671
AT-H_2-550-5	35.215	60.895	2.567	0	2.932	4.348	2.547	0.000	0.674
